# Supplementary material for: Effect of pre-exposure prophylaxis on risky sexual behaviour of female sex workers in Dakar, Senegal: A randomised controlled trial
Source: PLoS Med. 2025 Aug 18;22(8):e1004458. doi: 10.1371/journal.pmed.1004458 (PMC12407539; doi:10.1371/journal.pmed.1004458)
Supplement: S1 Trial Protocol — (S1_Trial_Protocol.DOCX) [file pmed.1004458.s001.docx]

**Title**

Effect of pre-exposure prophylaxis (PrEP) on risky sexual behaviours of female sex workers in Dakar, Senegal: A study protocol for a randomised controlled trial

**Abstract**

**Introduction:** PrEP (pre-exposure prophylaxis) is medicine people at risk for HIV take to prevent them from getting HIV. Senegal is in the process of rolling out PrEP gradually nationally. Female sex workers are one of the target groups for PrEP due to their high risk of contracting HIV. However, there is also a policy concern that taking PrEP might reduce their incentives for using a condom, especially when unprotected sex has higher renumeration. This may have public health consequences as condom use also curbs the transmission of other sexually-transmitted infections (STIs). Therefore, this study aims to study whether the drop in condom use is large enough to be of concern, and if possible, among which groups this drop is concentrated in.

**Methods and Analysis**: This study is a randomised controlled trial with two parallel groups. 1 Female sex workers aged 18 and over who took part in the July 2020 wave of a panel survey held in Dakar, Senegal who are still working in sex work at the point of that survey

**Ethics and Dissemination:** This trial has been prospectively registered with the ISRCTN Registry. We obtained ethical approval from Senegal National Ethics Committee and the UCL ethics committee before data collection. The findings of the trial will be published in peer-reviewed journals irrespective of the final results.

**Trial registration**:

ISRCTN16445862. Registered on 11 March 2021.

**Funding:**

This project is funded by a MRC grant awarded to Dr Aurélia Lépine MR/T00262X/1 and a grant from Erasmus university

**Keywords**

Randomised controlled trial; PrEP; vulnerable women; STIs/HIV

**Administrative information**

| **Title** | Effect of pre-exposure prophylaxis (PrEP) on risky sexual behaviours of female sex workers in Dakar, Senegal: A study protocol for a randomised controlled trial |
| --- | --- |
| **Trial registration** | ISRCTN16445862. Registered on 11 March 2021. |
| **Protocol version** | Version 1 |
| **Funding** | This project is funded by a MRC grant awarded to Dr Aurélia Lépine MR/T00262X/1 and a grant from Erasmus university |
| **Author details** | Wally TOH, Erasmus University  Aurelia LEPINE, UCL  Khady GUEYE, DSLI/MSAS  Mame Mor FALL, ANCS  Abdou Khoudia DIOP, DSLI/MSAS  El hadji Alioune MBAYE, DLSI  Cheikh Tidiane NDOUR, DLSI  Owen O'DONNELL, Erasmus University |
| **Name and contact information for the trial sponsor** | UK Research and Innovation 58 Victoria Embankment London EC4Y 0DS |
| **Role of sponsor** | The MRC had no role in the design of this study and will not have any role in any of these processes. |

**Introduction**

**Background and rationale {6a}**

When taken as prescribed, pre-exposure prophylaxis (PrEP) is highly effective in preventing HIV infection [1-2]. However, there is some concern that this protection may encourage risky sexual behaviour—*risk compensation—*that partially offsets the preventive effect [2-7]. For example, taking PrEP may reduce condom use or increase sexual activity with multiple partners. Such behaviours would increase the prevalence of other sexually-transmitted infections (STIs) that PrEP does not protect against.

Most research on behavioural responses to PrEP have focused on men who have sex with men (MSM). Evidence of risk compensation in this population is mixed [2, 8-10]. It is more evident in more recent studies [8-10]. This is consistent with an increasing behavioural response as awareness of PrEP’s effectiveness becomes more widespread [8-9].

Risk compensation could potentially be more prevalent among sex workers taking PrEP as they have an economic incentive to adopt riskier sexual behaviours. For example, they can charge a higher price for condomless sex [2, 11]. However, there is a lack of robust evidence on the phenomenon in this population. Observational studies have shown mixed evidence [7, 11, 13-14] that is difficult to interpret because sex workers who take PrEP have been observed to have riskier sexual behaviours and be at higher risk of STIs at baseline [16]. Qualitative research suggests that risk compensation may grow as sex workers become more convinced of the effectiveness of PrEP [12]. On the other hand, PrEP provision may bring hard-to-reach populations such as sex workers into closer contact with the health system, improving opportunities for routine STI testing and sexual health counselling [3].

In 2021/2022, Senegal rolled out PrEP to target high-risk populations, including female sex workers. Before the rollout, PrEP had been temporarily offered to only a limited cohort of female sex workersIn 2015, HIV/AIDS prevalence among female sex workers (6.6 percent) was nine times higher than the overall prevalence rate [18]. In Senegal, sex work is legal upon registration. Registered sex workers are required to visit a public health centre every month for health checks. Due to the strong stigma attached to sex work, many sex workers choose to remain unregistered.

This study aimed to use the rollout of PrEP to female sex workers in Senegal to obtain the first randomized experiment evidence for this high-risk population on risk compensation in response to PrEP.

**Objectives {7}**

Pre-exposure prophylaxis (PrEP) reduces the use of condoms among female sex workers.
2. PrEP reduces the perceived necessity of the use of condoms among female sex workers.

**Trial design {8}**

This study is a randomised controlled trial. We identified 500 respondents who were potentially eligible for PrEP and randomly assigned them into treatment and control groups (3:2). The randomization was stratified by (a) reported prior experience with PrEP in the 2015/16 PrEP pilot study, and (b) self-reported sexual risk-taking reported in the 2020 survey.

**Methods: Participants, interventions and outcomes**

**Study setting {9}**

PrEP initiation and follow-up for female sex workers (FSWs) was implemented by the Ministry of Health (MoH) and Social Action Senegal through public health facilities responsible for delivering prevention and treatment services mainly to registered sex workers and by the Alliance Nationale Contre le SIDA (ANCS) through community sites and mobile clinics that mainly target unregistered sex workers.

**Study participating centre**

Mobile clinic for PrEP and Survey location - Rufisque
Dakar
25000
Senegal

Mobile clinic for PrEP and Survey location - Pikine
Dakar
17000
Senegal

Mobile clinic for PrEP and Survey location - Mbao
Dakar
17000
Senegal

Survey location - Sebikotane
Dakar
20200
Senegal

**Eligibility criteria {10}**

Our sampling frame came from a survey of 604 FSWs conducted between June and August 2020 in Dakar, Senegal. The survey was the third wave of a cohort study, initiated in 2015 and followed-up in 2017, that interviewed FSWs who were at least 18 years old. In each survey wave, the sample was replenished with new participants who were recruited via snowball sampling by midwives at public health centres (registered sex workers) and sex worker facilitators (unregistered sex workers).

We identified participants who were potentially eligible for PrEP by excluding those who were not active in sex work in 2020 and those who were recorded as having a medical record of being HIV positive in any of the previous surveys. We identified 500 respondents who were potentially eligible for PrEP and randomly assigned them into treatment and control groups (3:2). The randomization was stratified by (a) reported prior experience with PrEP in the 2015/16 PrEP pilot study, and (b) self-reported sexual risk-taking reported in the 2020 survey.

**Who will take informed consent? {26a}**

Trained enumerators take informed consent by reading and explaining the information sheets (containing information on the study, objectives of the research, risks and benefits and explaining that participation is voluntary) to potential participants. Participants then have the opportunity to have an informed discussion with the enumerators regarding the study. Participants 21 years old and over are considered majors. Majors can offer their own consent to participate in the study. Adult representatives offering consent and all participants (minor and major) will receive a hard copy of the consent. An electronic copy, for researchers, of all consent forms (adult representative, minor, major) is stored electronically using an ODK-based survey.

**Additional consent provisions for collection and use of participant data and biological specimens {26b}**

Participants are informed during the informed consenting process that they will undergo biological. From October 2021 to January 2022, midwives and FSW peer facilitators working for MoH and ANCS approached potential participants in the treatment arm, informed of the availability of PrEP and asked if they were interested in receiving the medication. Interested participants continued to PrEP screening to establish whether each was still a FSW and satisfied medical criteria for PrEP eligibility: (a) good liver function assessed by creatinine level, (b) HIV negative, and (c) not pregnant. Those in the treatment group who eventually did not take up PrEP because they either were found to be ineligible or refused PrEP were not excluded from a survey used to measure outcomes. After this survey, potential PrEP candidates in the control arm were offered PrEP if they reported still being in sex work and they met the medical criteria. It is explained to participants that the reason we collect their data and biological specimens is to meet the research objective. Consent for biological specimens required for infection testing, using vaginal swabs and blood tests, is obtained during the consenting process.

**Interventions**

**The explanation for the choice of comparators {6b}**

The trial will compare participants who receive PrEP with participants who receive nothing.

**Intervention description {11a}**

From October 2021 to January 2022, midwives and FSW peer facilitators working for MoH and ANCS approached potential participants in the treatment arm, informed of the availability of PrEP and asked if they were interested in receiving the medication. Interested participants continued to PrEP screening to establish whether each was still a FSW and satisfied medical criteria for PrEP eligibility: (a) good liver function assessed by creatinine level, (b) HIV negative, and (c) not pregnant. Those in the treatment group who eventually did not take up PrEP because they either were found to be ineligible or refused PrEP were not excluded from a survey used to measure outcomes. After this survey, potential PrEP candidates in the control arm were offered PrEP if they reported still being in sex work and they met the medical criteria. At the end of the trial, PrEP was rolled out to the control group.

**Recruitment start date**

10/2021

**Recruitment end date**

01/2022

**Criteria for discontinuing or modifying allocated interventions {11b}**

All adverse events will be treated on a case-by-case basis. In the event of an unanticipated adverse event that may cause direct harm or threat to the participants or others that are related to study implementation. An unanticipated event report will be submitted promptly to the Senegal National Ethics Committee and the UCL ethics committee per protocol.

**Strategies to improve adherence to interventions {11c}**

The main strategies employed to encourage participant adherence to the intervention is to ensure that participants have the best possible experience whilst receiving PrEP. The community based organisation CBOs and specifically peer leaders and mobilisers support the adherence of participants to the intervention by keeping in regular contact with the participants throughout the lifetime of the project. Regular contact allows participants with concerns regarding the project or the intervention specifically the opportunity to speak up. Any problems are communicated to the research team and resolved. All resolutions are discussed with the participants. Peer leaders and mobilisers use their strong relationships with the participants and their status in this vulnerable population to engage participants and encourage adherence to the intervention.

**Relevant concomitant care permitted or prohibited during the trial {11d}**

There are no restrictions on concomitant care.

**Provisions for post-trial care {30}**

Any participants who suffer harm, e.g., psychological distress, as a consequence of the trial will be supported by the relevant CBO's psychological support services. Those in the control group, that remain in the study until the end of the project, will receive the same intervention at the end of the trial

**Outcomes {12}**

**Overview**

In April-May 2022, the midwives and FSW peer facilitators attempted to reach and interview in a survey all 500 candidates who had been randomized to the treatment or control arms. The survey covered PrEP usage, preventive health behaviours and details of the sex work undertaken. We had limited access to health system information on which participants eventually received PrEP medication. We relied predominantly on the self-reported survey data on PrEP usage. For those who reported using PrEP, this was cross-checked with midwives for registered sex workers and FSW peer facilitators for unregistered sex workers.

### Primary outcome measures

Measured with a survey expected to be held 3 months after the roll-out of PrEP:
1. Condom use with each of the last two clients and the last five clients measured via a combination of these methods:
1.1. Via direct questioning
1.2. Via list experiment
1.3. Via a newly-designed instrument aimed at eliciting individual condom use without compromising respondent confidentiality
2. Subjective perceptions of the necessity of condom use measured using a Likert-like scale

### Secondary outcome measures

Measured with a survey expected to be held 3 months after the roll-out of PrEP:
1. Number of occasional and regular clients seen in a fixed time frame, e.g. 7 days
2. Perceived HIV risk of clients
3. Price of each of the last two clients
4. Type of sex acts with last two clients/stayed overnight with last two clients
5. Earnings from sex work in a fixed time frame, e.g. 30 days
6. Household expenditure in a fixed time frame, e.g. 30 days
7. Food insecurity
8. Self-reported STI symptoms with last two clients
9. Mental health measured using the PHQ-9 questionnaire

**Participant timeline {13}**


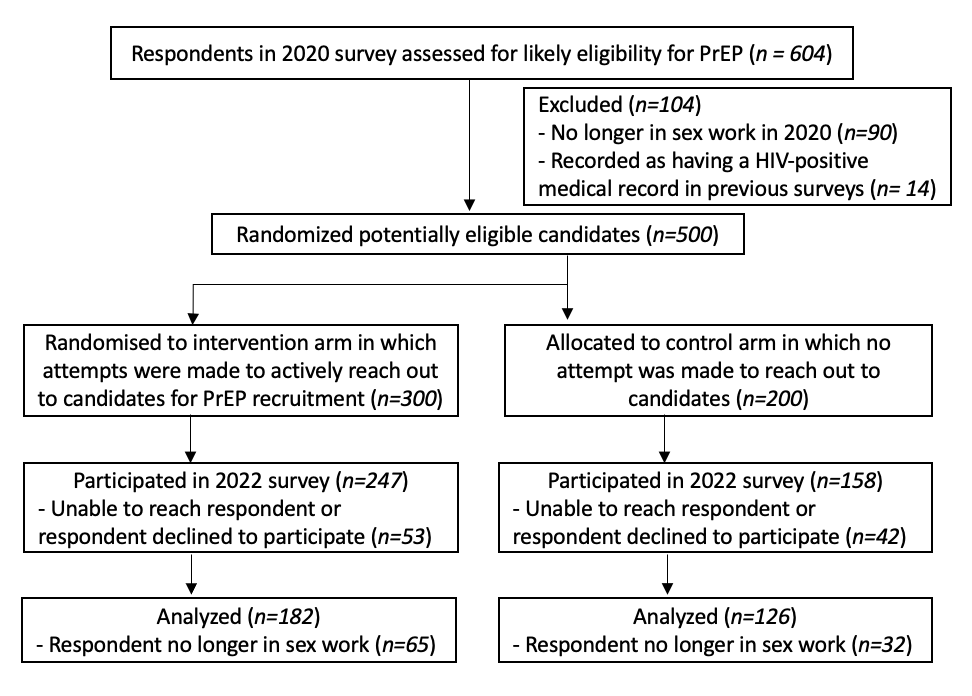


Figure 1: Schedule of enrolment, interventions, and assessments

**Sample size {14}**

Our sampling frame came from a survey of 604 FSWs conducted between June and August 2020 in Dakar, Senegal. The survey was the third wave of a cohort study, initiated in 2015 and followed-up in 2017, that interviewed FSWs who were at least 18 years old. In each survey wave, the sample was replenished with new participants who were recruited via snowball sampling by midwives at public health centres (registered sex workers) and sex worker facilitators (unregistered sex workers).

We identified participants who were potentially eligible for PrEP (Figure 1) by excluding those who were not active in sex work in 2020 and those who were recorded as having a medical record of being HIV positive in any of the previous surveys. We identified 500 respondents who were potentially eligible for PrEP and randomly assigned them into treatment and control groups (3:2).

**Recruitment {15}**

In April-May 2022, the midwives and FSW peer facilitators attempted to reach and interview in a survey all 500 candidates who had been randomized to the treatment or control arms. The survey covered PrEP usage, preventive health behaviours and details of the sex work undertaken. We had limited access to health system information on which participants eventually received PrEP medication. We relied predominantly on the self-reported survey data on PrEP usage. For those who reported using PrEP, this was cross-checked with midwives for registered sex workers and FSW peer facilitators for unregistered sex workers.

**Assignment of interventions: allocation**

We used computer based randomisation and allocated participants in the group. We then sent lists of participants to recruit to the implementing CBOs and health facilities to recruit participants. Only participants on the list were eligible to PrEP.

**Assignment of interventions: Blinding**

**Who will be blinded {17a}**

Participant allocation to the treatment arm is known by the participants, the enumerators and supervisors. However, data analysts will be blinded during the data analysis period.

**Data collection and management**

**Plans for assessment and collection of outcomes {18a}**

Outcomes are measured via the biobehavioural and socioeconomic survey. The survey questionnaire will include sociodemographic questions, income, health status, demand for healthcare, economic shocks, STI prevention, risky sexual behaviours, violence, personality traits and preferences.

**Plans to promote participant retention and complete follow-up {18b}**

**Participant retention**

Participants are not incentivised or coerced to participate in the study. During the informed consent process, the benefits of the study are outlined in detail to encourage participant retention. The primary benefit highlighted is the possibility that this project will contribute to the proposal of public policy intervention to improve the quality of life of young women who engage in transactional and commercial sex.

In addition, all participants receive compensation for their time and travel costs. Participants are compensated 2000 FCFA for their time and travel when responding to on-site questionnaires. This compensation will act to reduce any barriers to participant participation (e.g., transport fees).

To retain participants in the control group, the project offers the same intervention to all eligible control group participants at the end of the data collection period.

**Participant withdrawal**

Participation is voluntary and participants can withdraw at any time, without giving reasons, by notifying a project staff member. Participant information already collected will be suppressed from the dataset. Withdrawal of participants on the treatment arm of the study will not impact any services or medical treatment they receive or any relationships they have with those treating them outside of the project.

**Data management {19}**

Socioeconomic questionnaires will be administered by enumerators in the field. All potential participants will be screened before recruitment onto the study to ensure they have not already participated. Participant responses are entered directly into an ODK-based questionnaire using Android tablets and uploaded to the ODK secure server in the UK when a 3G/WIFI connection is available (immediately, or at the end of each day). On survey completion, the data will be transferred to the UCL Secure Server, a fully encrypted (SSL) server with a direct data link to the android device. The ODK-based questionnaire is programmed with a logic that minimises missing data (e.g., enumerators will not be able to skip questions) and data quality (e.g., range checks will be conducted regularly). Any issues regarding data quality will be reported to the Principal investigator (PI) and directly to the enumerators and the team in the field.

All data will be stored in accordance with University College London's data storage policies.

After completion of the project, data will be made openly available through the UCL data repository, subject to the ability to remove personally identifiable information. Data will be deposited with UCL research data repository (<https://www.ucl.ac.uk/library/research-support/research-data-management/ucl-research-data-repository>), where it will be given a Digital Object Identifier (DOI). The DOI will be cited in project reports and journal publications through a Data Access Statement or citation list. Metadata will also be made available to 3rd party research catalogues, such as https://datamed.org/.

Anonymised data will be made openly available using a permissive licence, such as Creative Commons Attribution (CC-BY). If complete anonymity cannot be provided, it will be made available through a controlled access system. If data must be made available via controlled access, interested parties will be asked to provide information on their research (purpose, institutional affiliation, ethics approval) and sign a data sharing agreement indicating they will comply with the consent form and will not attempt to re-identify individuals. Access requests will be evaluated by the project team in the first instance. In cases where an access request is denied and the requester wishes to appeal, the request will be escalated to the UCL Research Governance Committee for consideration.

**Confidentiality {27}**

The following measures have been put in place to ensure data confidentiality and to respect the privacy of participants:

1. Implementation of and compliance with the code of ethics

All study staff, inclusive of hired and trained experienced local enumerators and interviewers, must sign a commitment to comply with the code of ethics. Research partners with access to data have signed a sub-contract with a clause which ensures that all data collected must remain confidential.

1. Participation is confidential

Socioeconomic and biobehavioural data collection will be conducted in a private location at the CBOs or health facility. Participants must not share a telephone to be enrolled in the SMS survey..

1. Secure storage of data

During the trial, all paper-based records will be kept securely in a locked cabinet, in a locked office, and securely transferred to the Ministry of Health office where they will be transferred to a centralized, 128-bit encrypted and password-protected database. All electronic data will be stored on the UCL Secure Server.

1. Aggregation and anonymisation of data

Individual responses will be deidentified and aggregated for analysis and reporting. All published findings, including any quotes used, will not allow for individuals to be identified. All results will be presented in aggregate form.

**Plans for collection, laboratory evaluation and storage of biological specimens for genetic or molecular analysis in this trial/future use {33}**

N/A

**Statistical methods**

**Statistical methods for primary and secondary outcomes {20a}**

All statistical analyses will be conducted using STATA. Descriptive statistics will first be used to summarise participant characteristics between the study groups. To investigate the effect of health insurance on primary and secondary outcomes, the analysis will use an intention-to-treat (ITT) approach where participants will be analysed in their allocated randomised study group. If there are non-compliers, we will also estimate the local average treatment effect (LATE). Outcomes for the treatment versus the control group will then be compared accounting for any baseline or attrition differences using ordinary least squared.

**Interim analyses {21b}**

No interim analysis will be conducted.

**Methods for additional analyses (e.g., subgroup analyses) {20b}**

Further analysis will investigate if the effect of PrEP varies depending on the factors used for stratification.

**Methods in analysis to handle protocol non-adherence and any statistical methods to handle missing data {20c}**

Non-compliance issues will be investigated by estimating the local average treatment effect (LATE).

**Plans to give access to the full protocol, participant-level data and statistical code {31c}**

Data and statistical code will be made openly available through the UCL data repository, subject to the ability to remove personally identifiable information. Data will be deposited with UCL research data repository (<https://www.ucl.ac.uk/library/research-support/research-data-management/ucl-research-data-repository>), where it will be given a Digital Object Identifier (DOI). The DOI will be cited in project reports and journal publications through a Data Access Statement or citation list. Metadata will also be made available to 3rd party research catalogues, such as <https://datamed.org/>. Qualitative responses will not be uploaded to this repository due to the potential to identify participants.

Anonymised data will be made openly available using a permissive licence, such as Creative Commons Attribution (CC-BY). If complete anonymity cannot be provided, it will be made available through a controlled access system. If data must be made available via controlled access, interested parties will be asked to provide information on their research (purpose, institutional affiliation, ethics approval) and sign a data sharing agreement indicating they will comply with the consent form and will not attempt to re-identify individuals. Access requests will be evaluated by the project team in the first instance. In cases where an access request is denied and the requester wishes to appeal, the request will be escalated to the UCL Research Governance Committee for consideration.

**Oversight and monitoring**

**Composition of the coordinating centre and trial steering committee {5d}**

There is no formal committee for the coordination and oversight of this trial. The PI will oversee and coordinate all aspects of the trial with the support of the project’s researchers. All decisions impacting the project protocol will be discussed with the lead researchers. The Advisory Group made up of experts internal (project staff from UCL and local partners (Ministry of Health of Senegal through DLSI and ANCS) will be consulted at key decision points.

**Data monitoring {21a}**

There is no formal committee for monitoring data collected as part of the project. Data monitoring will be undertaken by a researcher supervised by the PI. Questionnaire responses will be checked daily to determine any problems with the data collection instruments.

**Adverse event reporting and harms {22}**

An adverse event will be defined as a participant becoming significantly distressed as a consequence of their involvement in the trial. All adverse events, as a consequence of participation in the study, occurring after the entry into the study and until the end of the study will be recorded and will be treated on a case-by-case basis. In the event of an unanticipated adverse event that may cause direct harm or threat to the participants or others that are related to study implementation, the local staff will notify the PI. Per protocol, an unanticipated event report will be submitted promptly to the Senegal National Ethics Committee and the UCL ethics committee.

During the study, we might have some minor adverse events such as syncope during blood drawing for laboratory testing. Patients will be informed of this risk. Laboratory technicians will be trained on how to deal with these minor events. An unlikely adverse event could be an infection at the site of the needle prick, the chance of which will be minimized through observing international standards of blood sample collection. The respondent will be properly counselled on how, where and when to seek treatment in case of such an incident. The use of PrEP will be closely monitored by medical doctors involved in the PrEP rolled out in Senegal.

**Frequency and plans for auditing trial conduct {23}**

The PI meets weekly with researchers conducting the daily activities of the trial online to discuss and review trial progress, and at least monthly with the full investigator team. Any adverse events or issues in trial progress will be promptly communicated with the investigator team at these meetings or between meetings if necessary. Progress to the trial funder will be reported annually. Any trial protocol amendments will be reported to the University College London Research Ethics Committee and National Ethics Committee in Senegal.

**Plans for communicating important protocol amendments to relevant parties (e.g., trial participants, ethical committees) {25}**

Any amendments to the protocol will be provided to the University College London Research Ethics Committee and National Ethics Committee in Senegal.

**Dissemination plans {31a}**

The project partners ANCS and the Ministry of Health in Senegal, and the funders will be provided with a summary of the findings of the trial. The findings will also be reported in high-impact scientific journal articles and presented at scientific national and international conferences and in public forums as requested.

A policy brief will also be disseminated by those stakeholders through internal newsletters.

**Abbreviations**

- PrEP – Pre-exposure prophylaxis
- ANCS – Alliance Nationale des communautes pour la sante
- DLSI – Division de lutte contre le SIDA et les IST, Ministère de la sante du Senegal
- UCL – University College London
- DSH – Data Safe Haven
- CBO – Community-based organisation
- STI – Sexually transmitted infection
- HIV – Human immunodeficiency virus
- CEO – Chief Executive Officer
- PI – Principal investigator
- UKRI – UK Research and Innovation Council

**Declarations**

**Acknowledgements**

We would like to say thank study participants.

**Authors' contributions {31b}**

WT led the design development, followed data collection, analysed the data, led the drafting of the manuscript, contributed to the design of the study. AL conceived of and co-led the design of the study, led the proposal and the protocol development, contributed to the drafting of the manuscript and gave the final approval of the manuscript. All authors read and approved the final manuscript.

**Funding {4}**

This project is funded by a MRC grant awarded to Dr Aurélia Lépine MR/T00262X/1 and a grant from Erasmus university. UKRI has no role in the design or conduct of the study or the interpretation and dissemination of the findings. UKRI may be contacted at [communications@ukri.org](mailto:communications@ukri.org) or <https://www.ukri.org/about-us/contact-us/>.

**Availability of data and material {29}**

The team at UCL and Erasmus will have access to the final dataset, held on the DSH at UCL. The data will be available on the UCL repository at the time of publication of the trial results.

**Consent for publication {32}**

Not applicable.

**Competing interests {28}**

The authors declare that they have no competing interests.

**References**

[1] PrEP effectiveness [Internet]. Centers for Disease Control and Prevention (CDC). 2022 [cited 2023Jan26]. Available from: <https://www.cdc.gov/hiv/basics/prep/prep-effectiveness.html>

[2] Murchu EO, Marshall L, Teljeur C, Harrington P, Hayes C, Moran P, Ryan M. Oral pre-exposure prophylaxis (PrEP) to prevent HIV: a systematic review and meta-analysis of clinical effectiveness, safety, adherence and risk compensation in all populations. BMJ open. 2022 May 1;12(5):e048478.

[3] Rojas Castro D, Delabre RM, Molina JM. Give PrEP a chance: moving on from the “risk compensation” concept. Journal of the International AIDS society. 2019 Aug;22:e25351.

[4] Peltzman S. The effects of automobile safety regulation. Journal of political Economy. 1975 Aug 1;83(4):677-725.

[5] Wilson NL, Xiong W, Mattson CL. Is sex like driving? HIV prevention and risk compensation. Journal of Development Economics. 2014 Jan 1;106:78-91.

[6] Carlo Hojilla J, Koester KA, Cohen SE, Buchbinder S, Ladzekpo D, Matheson T, Liu AY. Sexual behavior, risk compensation, and HIV prevention strategies among participants in the San Francisco PrEP demonstration project: a qualitative analysis of counseling notes. AIDS and Behavior. 2016 Jul;20:1461-9.

[7] Giguère, K., Béhanzin, L., Guédou, F. A., Talbot, D., Leblond, F. A., Goma-Matsétsé, E., ... & Alary, M. (2019). PrEP use among female sex workers: no evidence for risk compensation. Journal of acquired immune deficiency syndromes (1999), 82(3), 257.

[8] Powell VE, Gibas KM, DuBow J, Krakower DS. Update on HIV preexposure prophylaxis: Effectiveness, drug resistance, and risk compensation. Current infectious disease reports. 2019 Aug;21(8):1-8.

[9] Traeger MW, Schroeder SE, Wright EJ, Hellard ME, Cornelisse VJ, Doyle JS, Stoové MA. Effects of pre-exposure prophylaxis for the prevention of human immunodeficiency virus infection on sexual risk behavior in men who have sex with men: a systematic review and meta-analysis. Clinical Infectious Diseases. 2018 Aug 16;67(5):676-86.

[10] Yan X, Jia Z, Zhang B. Evaluating the risk compensation of HIV/AIDS prevention measures. The Lancet Infectious Diseases. 2022 Apr 1;22(4):447-8.

[11] Quaife M, Vickerman P, Manian S, Eakle R, Cabrera‐Escobar MA, Delany‐Moretlwe S, Terris‐Prestholt F. The effect of HIV prevention products on incentives to supply condomless commercial sex among female sex workers in South Africa. Health economics. 2018 Oct;27(10):1550-66.

[12] Kayesu I, Mayanja Y, Nakirijja C, Machira YW, Price M, Seeley J, Siu G. Uptake of and adherence to oral pre-exposure prophylaxis among adolescent girls and young women at high risk of HIV-infection in Kampala, Uganda: A qualitative study of experiences, facilitators and barriers. BMC women's health. 2022 Dec;22(1):1-4.

[13] Bowring AL, Ampt FH, Schwartz S, Stoové MA, Luchters S, Baral S, Hellard M. HIV pre‐exposure prophylaxis for female sex workers: ensuring women’s family planning needs are not left behind. Journal of the International AIDS Society. 2020 Feb;23(2):e25442.

[14] Mboup A, Béhanzin L, Guédou FA, Geraldo N, Goma‐Matsétsé E, Giguère K, Aza‐Gnandji M, Kessou L, Diallo M, Kêkê RK, Bachabi M. Early antiretroviral therapy and daily pre‐exposure prophylaxis for HIV prevention among female sex workers in Cotonou, Benin: a prospective observational demonstration study. Journal of the International AIDS Society. 2018 Nov;21(11):e25208.

[15] Guest G, Shattuck D, Johnson L, Akumatey B, Clarke EE, MacQUEEN KM. Changes in sexual risk behavior among participants in a PrEP HIV prevention trial. Sexually transmitted diseases. 2008 Dec 1:1002-8.

[16] Grant RM, Anderson PL, McMahan V, Liu A, Amico KR, Mehrotra M, Hosek S, Mosquera C, Casapia M, Montoya O, Buchbinder S. An observational study of preexposure prophylaxis uptake, sexual practices, and HIV incidence among men and transgender women who have sex with men. The Lancet. Infectious diseases. 2014 Sep;14(9):820.

[17] Senegal [Internet]. Unaids.org. [cited 2023 Dec 2]. Available from: <https://www.unaids.org/en/regionscountries/countries/senegal>

[18] APAPS & IRESSEF (2016). Enquête nationale de surveillance combinée desinfections sexuallement transmissibles et du VIH/SIDA, Groupe cible: Travailleuses du sexe. Dakar, Senegal.
