# Supplementary material for: Effect of pre-exposure prophylaxis on risky sexual behaviour of female sex workers in Dakar, Senegal: A randomised controlled trial
Source: PLoS Med. 2025 Aug 18;22(8):e1004458. doi: 10.1371/journal.pmed.1004458 (PMC12407539; doi:10.1371/journal.pmed.1004458)
Supplement: S1 Consort Checklist — (DOCX) [file pmed.1004458.s002.docx]

| **Section/topic** | **No** | **CONSORT 2025 checklist item description** | **Reported on page no.** |
| --- | --- | --- | --- |
| **Title and abstract** |  |  |  |
| Title and structured abstract | 1a | Identification as a randomised trial | Title page |
|  | 1b | Structured summary of the trial design, methods, results, and conclusions | Abstract |
| **Open science** |  |  |  |
| Trial registration | 2 | Name of trial registry, identifying number (with URL) and date of registration | Methods and Findings – Study design and participants – Paragraph 1 |
| Protocol and analysis plan | 3 | Where the trial protocol and statistical analysis plan can be accessed | Protocol submitted as supplementary info |
| Data sharing | 4 | Where and how the individual de-identified participant data, statistical code and any other materials can be accessed | UCL repository (under review) |
| Funding and conflicts of interest | 5a | Sources of funding and role of funders | Methods and Findings – Role of the funding source – Paragraph 1 |
|  | 5b | Financial and other conflicts of interest of the manuscript authors | Role of the funding source – Paragraph 1 |
| **Introduction** |  |  |  |
| Background and rationale | 6 | Scientific background and rationale | Background – Paragraphs 1–3 |
| Objectives | 7 | Specific objectives related to benefits and harms | Background – Paragraph 4 |
| **Methods** |  |  |  |
| Patient and public involvement | 8 | Details of patient or public involvement in the design, conduct and reporting | Not applicable |
| Trial design | 9 | Trial type (e.g., parallel, crossover), allocation ratio, framework | Methods and Findings – Study design and participants – Paragraph 1; Randomization and masking – Paragraph 1 |
| Changes to trial protocol | 10 | Important post-commencement changes and reasons | Methods and Findings – Outcomes – Paragraph 4; Conclusions – Limitations – Paragraph 3 |
| Trial setting | 11 | Settings and locations of trial | Methods and Findings – Procedures – Paragraph 4 |
| Eligibility criteria | 12a | Eligibility criteria for participants | Methods and Findings – Study design and participants – Paragraph 3 |
|  | 12b | Eligibility criteria for sites/intervention providers (if applicable) | Not applicable |
| Intervention and comparator | 13 | Intervention and comparator description, and access to detailed materials | Methods and Findings – Procedures – Paragraph 1 |
| Outcomes | 14 | Prespecified outcomes (variables, metrics, time points, aggregation) | Methods and Findings – Outcomes – Paragraphs 1–3 |
| Harms | 15 | How harms were defined and assessed | Procedures – Paragraph 3 |
| Sample size | 16a | How determined and assumptions used | Methods and Findings – Outcomes – Last paragraph |
|  | 16b | Interim analyses and stopping guidelines (if applicable) | Not applicable |
| **Randomisation** |  |  |  |
| Sequence generation | 17a | Who and how random allocation sequence was generated | Supplementary Text S1 – Implementation of stratified randomization |
|  | 17b | Type of randomisation, restrictions used | Supplementary Text S1 – Implementation of stratified randomization |
| Allocation concealment mechanism | 18 | Allocation concealment methods and details | Supplementary Text S1 – Implementation of stratified randomization |
| Implementation | 19 | Who enrolled, assigned participants and had access to the sequence | Methods and Findings – Randomization and Masking – Paragraph 1 |
| Blinding | 20a | Who was blinded and how | Methods and Findings – Randomization and Masking – Paragraph 2 |
|  | 20b | Similarity of interventions (if relevant) | Not applicable |
| Statistical methods | 21a | Methods for primary and secondary outcome comparison | Methods and Findings – Statistical analysis – All paragraphs |
|  | 21b | Definition of analysis population and groups | Methods and Findings – Statistical analysis – Paragraphs 1–2 |
|  | 21c | How missing data were handled | Methods and Findings – Statistical analysis – Paragraph 4 |
|  | 21d | Additional analyses (subgroup, sensitivity) and whether prespecified | Methods and Findings – Statistical analysis – Paragraph 5 |
| **Results** |  |  |  |
| Participant flow | 22a | Numbers assigned, treated, analysed for primary outcome | Methods and Findings – Results – Paragraph 1; Figure 1 |
|  | 22b | Post-randomisation losses/exclusions and reasons | Methods and Findings – Results – Paragraph 1; Figure 1 |
| Recruitment | 23a | Dates of recruitment and follow-up | Methods and Findings – Procedures – Paragraphs 1 & 3 |
|  | 23b | Why the trial ended or was stopped | Not applicable |
| Intervention and comparator delivery | 24a | Description of intervention as delivered | Methods and Findings – Procedures – Paragraph 1 |
|  | 24b | Concomitant care received | Not reported |
| Baseline data | 25 | Baseline demographic/clinical characteristics table | Methods and Findings – Results – Paragraph 2; Table 1 |
| Numbers analysed, outcomes, estimation | 26 | Participants analysed per group, available data, outcome results, effect sizes, CIs | Methods and Findings – Results – Paragraphs 5–9 |
| Harms | 27 | All important harms or unintended effects | Procedures – Paragraph 3 |
| Ancillary analyses | 28 | Subgroup/sensitivity analyses, prespecified or exploratory | Conclusions – Results – Paragraph 9 |
| **Discussion** |  |  |  |
| Interpretation | 29 | Interpretation aligned with results and relevant evidence | Conclusions – Discussion – All paragraphs; Conclusions – Summary – All paragraphs |
| Limitations | 30 | Limitations including bias, imprecision, generalisability, multiplicity | Conclusions – Limitations – All paragraphs |

Citation: Hopewell S, Chan AW, Collins GS, Hróbjartsson A, Moher D, Schulz KF, et al. CONSORT 2025 Statement: updated guideline for reporting randomised trials. BMJ. 2025; 388:e081123. <https://dx.doi.org/10.1136/bmj-2024-081123>
© 2025 Hopewell et al. This is an Open Access article distributed under the terms of the Creative Commons Attribution License (<https://creativecommons.org/licenses/by/4.0/>), which permits unrestricted use, distribution, and reproduction in any medium, provided the original work is properly cited.

*We strongly recommend reading this statement in conjunction with the CONSORT 2025 Explanation and Elaboration and/or the CONSORT 2025 Expanded Checklist for important clarifications on all the items. We also recommend reading relevant CONSORT extensions. See [www.consort-spirit.org](http://www.consort-spirit.org).
