# Supplementary material for: Effect of pre-exposure prophylaxis on risky sexual behaviour of female sex workers in Dakar, Senegal: A randomised controlled trial
Source: PLoS Med. 2025 Aug 18;22(8):e1004458. doi: 10.1371/journal.pmed.1004458 (PMC12407539; doi:10.1371/journal.pmed.1004458)
Supplement: S1 SPIRIT Checklist — (DOCX) [file pmed.1004458.s003.docx]

**SPIRIT 2025 checklist of items to address in a randomized trial protocol***

| **Section / Topic** | **No** | **SPIRIT 2025 checklist item description** | **Reported on page no.** |
| --- | --- | --- | --- |
| **Administrative information** |  |  |  |
| Title and structured summary | 1a | Title stating the trial design, population, and interventions, with identification as a protocol | Title page |
|  | 1b | Structured summary of trial design and methods, including items from WHO Trial Registration Data Set | Abstract |
| Protocol version | 2 | Version date and identifier | Title page footer |
| Roles and responsibilities | 3a | Names, affiliations, and roles of protocol contributors | Title page; Supplementary Info |
|  | 3b | Name and contact information for the trial sponsor | Methods and Findings – Role of the funding source – Paragraph 1 |
|  | 3c | Role of trial sponsor and funders in trial design, conduct, analysis, reporting | Methods and Findings – Role of the funding source – Paragraph 1 |
|  | 3d | Composition, roles, responsibilities of coordinating site, steering committee, etc., if applicable | Not applicable |
| **Open science** |  |  |  |
| Trial registration | 4 | Name of trial registry, number (with URL), and date of registration | Methods and Findings – Study design and participants – Paragraph 1 |
| Protocol and SAP access | 5 | Where protocol and statistical analysis plan can be accessed | Protocol submitted as supplementary info |
| Data sharing | 6 | Where and how participant data, statistical code, and materials will be accessible | UCL repository (under review) |
| Funding and conflicts of interest | 7a | Sources of funding and support | Methods and Findings – Role of the funding source – Paragraph 1 |
|  | 7b | Financial and other conflicts of interest for PIs and committee members | Methods and Findings – Role of the funding source – Paragraph 1 |
| Dissemination policy | 8 | Plans to communicate trial results to participants, public, and other stakeholders | Protocol – Dissemination plan; Trial registration record |
| **Introduction** |  |  |  |
| Background and rationale | 9a | Scientific background and rationale, including relevant studies | Background – Paragraphs 1–3 |
|  | 9b | Explanation for choice of comparator | Background – Paragraph 4 |
| Objectives | 10 | Specific objectives related to benefits and harms | Background – Paragraph 4 |
| **Methods: Patient and public involvement, trial design** |  |  |  |
| Patient and public involvement | 11 | Plans for patient/public involvement in design, conduct, reporting | Not applicable |
| Trial design | 12 | Description of trial design including type, allocation ratio, and framework | Methods and Findings – Study design and participants – Paragraph 1 |
| **Methods: Participants, interventions, outcomes** |  |  |  |
| Trial setting | 13 | Settings and locations of the trial | Methods and Findings – Procedures – Paragraph 4 |
| Eligibility criteria | 14a | Eligibility criteria for participants | Methods and Findings – Study design and participants – Paragraph 3 |
|  | 14b | Eligibility for sites and providers | Not applicable |
| Intervention and comparator | 15a | Description with replication detail; access to materials | Methods and Findings – Procedures – Paragraph 1 |
|  | 15b | Criteria for modifying intervention/comparator | Methods and Findings – Procedures – Paragraph 3 |
|  | 15c | Strategies to improve and monitor adherence | Methods and Findings – Procedures – Paragraph 3 |
|  | 15d | Concomitant care permitted/prohibited | Not reported |
| Outcomes | 16 | Primary and secondary outcomes with definitions, metrics, aggregation, and time points | Methods and Findings – Outcomes – Paragraphs 1–3 |
| Harms | 17 | How harms are defined and will be assessed | Procedures – Paragraph 3 |
| Participant timeline | 18 | Schedule of enrolment, intervention, assessment – schematic recommended | Figure 1; Methods and Findings – Study design and participants |
| Sample size | 19 | How sample size was determined including assumptions | Methods and Findings – Outcomes – Last paragraph |
| Recruitment | 20 | Strategies to achieve target sample size | Methods and Findings – Study design and participants – Paragraph 2 |
| **Methods: Assignment of interventions** |  |  |  |
| Sequence generation | 21a | Who and how random sequence was generated | Supplementary Text S1 – Implementation of stratified randomization |
|  | 21b | Type of randomization and restrictions | Supplementary Text S1 – Implementation of stratified randomization |
| Allocation concealment mechanism | 22 | How sequence was implemented and concealed | Supplementary Text S1 – Implementation of stratified randomization |
| Implementation | 23 | Who will enroll, assign participants and access sequence | Methods and Findings – Randomization and Masking – Paragraph 1 |
| Blinding | 24a | Who will be blinded | Methods and Findings – Randomization and Masking – Paragraph 2 |
|  | 24b | How blinding will be achieved, similarity of interventions | Not applicable |
|  | 24c | When unblinding is permissible and how | Methods and Findings – Randomization and Masking – Paragraph 2 |
| **Methods: Data collection, management, and analysis** |  |  |  |
| Data collection methods | 25a | Plans for data collection, quality control, and instruments | Methods and Findings – Procedures – Paragraph 4 |
|  | 25b | Plans for participant retention and data for those who discontinue | Methods and Findings – Procedures – Paragraph 4 |
| Data management | 26 | Plans for data entry, coding, security, storage | Supplementary Materials – Data management plan |
| Statistical methods | 27a | Statistical methods for primary, secondary, and harms | Methods and Findings – Statistical analysis – All paragraphs |
|  | 27b | Who will be included in each analysis and grouping | Methods and Findings – Statistical analysis – Paragraphs 1–2 |
|  | 27c | Handling of missing data | Methods and Findings – Statistical analysis – Paragraph 4 |
|  | 27d | Additional analyses (e.g., sensitivity, subgroup) | Methods and Findings – Statistical analysis – Paragraph 5 |
| **Methods: Monitoring** |  |  |  |
| Data monitoring committee | 28a | DMC composition, role, independence, conflicts of interest | Not applicable – no DMC |
| Interim analyses | 28b | Interim analyses and stopping rules | Not applicable |
| Trial monitoring | 29 | Monitoring procedures or rationale for no monitoring | Not monitored – small trial |
| **Ethics** |  |  |  |
| Research ethics approval | 30 | Plans for seeking REC/IRB approval | Methods and Findings – Ethics approval section |
| Protocol amendments | 31 | Plans for communicating protocol modifications | Ethics and dissemination section of protocol |
| Consent or assent | 32a | Who will obtain consent and how | Methods and Findings – Procedures – Paragraph 2 |
|  | 32b | Consent for additional data/biological specimens | Not applicable |
| Confidentiality | 33 | How confidentiality will be maintained | Methods and Findings – Procedures – Paragraph 2; Data management plan |
| Ancillary and post-trial care | 34 | Provisions for post-trial care or harm compensation | Ethics and dissemination section of protocol |

*We strongly recommend reading this checklist in conjunction with the SPIRIT 2025 Explanation and Elaboration and the SPIRIT 2025 Expanded Checklist for important clarifications on all the items. We also recommend reading relevant SPIRIT extensions. See [www.consort-spirit.org](http://www.consort-spirit.org)

Citation: Chan A-W, Boutron I, Hopewell S, Moher D, Schulz KF, et al. SPIRIT 2025 statement: updated guideline for protocols of randomised trials. BMJ 2025;389:e081477. <https://dx.doi.org/10.1136/bmj-2024-081477>

© 2025 Chan A-W et al. This is an Open Access article distributed under the terms of the Creative Commons Attribution License (<https://creativecommons.org/licenses/by/4.0/>), which permits unrestricted use, distribution, and reproduction in any medium, provided the original work is properly cited.
