# Supplementary material for: Effect of pre-exposure prophylaxis on risky sexual behaviour of female sex workers in Dakar, Senegal: A randomised controlled trial
Source: PLoS Med. 2025 Aug 18;22(8):e1004458. doi: 10.1371/journal.pmed.1004458 (PMC12407539; doi:10.1371/journal.pmed.1004458)
Supplement: S1 File — Text A: Stratified randomisation. Text B: List experiment method. Text C: Colorbox method. Table A: Survey questions used to derive outcomes. Table B: Pre-attrition cohort characteristics by treatment status, at baseline (2020). Table C: Probability of analysis cohort inclusion by treatment assignment and baseline (2020) characteristics (n = 500). Table D: Characteristics of analysis cohort in 2020 and at endline (2022) (n = 308). Table E: Outcome means for control and treatment groups at endline. Table F: Mean predicted outcomes under counterfactual of no PrEP use. Table G: Unadjusted risk differences (RDs) in condom use by PrEP referral and PrEP use. Table H: Adjusted risk differences (RDs) in condom use by PrEP referral and PrEP use—robustness to reweighting analysis cohort and treatment group. Table I: Adjusted risk differences (RDs) in condom use by PrEP referral and PrEP use—alternative measurements of condom use. Table J: Adjusted mean differences (MDs) in secondary outcomes and HIV/STI risk perceptions by PrEP referral and use—alternative specifications. (S1_File.DOCX) [file pmed.1004458.s004.docx]

**Effect of pre-exposure prophylaxis on risky sexual behaviour of female sex workers in Dakar, Senegal: A randomised controlled trial**

**Supplementary materials**

Table of Contents

[Text A. Stratified randomisation 2](#_Toc195482616)

[Text B: List experiment method 4](#_Toc195482617)

[Text C: Colorbox method 6](#_Toc195482618)

[Table A: Survey questions used to derive outcomes 9](#_Toc195482619)

[Table B: Pre-attrition cohort characteristics by treatment status, at baseline (2020) 11](#_Toc195482620)

[Table C: Probability of analysis cohort inclusion by treatment assignment and baseline (2020) characteristics (n=500) 13](#_Toc195482621)

[Table D: Characteristics of analysis cohort in 2020 and at endline (2022) (n=308) 14](#_Toc195482622)

[Table E: Outcome means for control and treatment groups at endline 15](#_Toc195482623)

[Table F: Mean predicted outcomes under counterfactual of no PrEP use 16](#_Toc195482624)

[Table G: Unadjusted risk differences (RDs) in condom use by PrEP referral and use 17](#_Toc195482625)

[Table H: Adjusted risk differences in condom use by PrEP referral and use – robustness to reweighting analysis cohort and treatment group 18](#_Toc195482626)

[Table I: Adjusted risk differences in condom use by PrEP referral and use – alternative measurements of condom use 19](#_Toc195482627)

[Table J: Adjusted mean differences (MDs) in secondary outcomes and HIV/STI risk perceptions by PrEP referral and use – alternative specifications 20](#_Toc195482628)

# Text A. Stratified randomisation

Randomisation was stratified by reported prior experience with pre-exposure prophylaxis (PrEP) and sexual risk taking.

**Definition of Prior PrEP experience**

In the 2017 and 2020 surveys, participants were asked whether they had participated in a 2015/16 PrEP pilot study. If, in either survey, they reported having participated in the pilot, then they were classified as having prior PrEP experience.

**Definition of Sexual risk taking**

In the 2020 survey, participants were asked: “When it comes to your attitude to risk, how would you place yourself on a scale from 0 to 10. 0 corresponds to very cautious people who try to limit the risks of life. 10 corresponds to the most adventurous people who like to take risks. With regard to your sexual behavior, how would you rate the risks you take?”. We treated low-risk participants as those who gave an answer of 2 and below, and the rest as high-risk participants.

**Strata definition**

4 strata were created from the 2 variables:

- Prior PrEP experience + Low self-reported sexual risk-taking
- No prior PrEP experience + Low self-reported sexual risk-taking
- Prior PrEP experience + High self-reported sexual risk-taking
- No prior PrEP experience + High self-reported sexual risk-taking

**Implementation of stratified randomization**

A random number was generated using STATA. The data was ordered on this random number. Next, within each stratum, a running variable was created, 1 for the first observation, 2 for the second, … N for the n-th observation. The desired control proportion was 40%. Hence, a cutoff of each stratum was calculated as 40% * N. For each stratum, observations with a running variable value before this stratum-specific cutoff were treated as control, and the rest as treated.

The resultant balance per stratum is shown in **Table S1.1** below.

**Table S1.1 Balance of strata across randomized groups**

|  | **Treatment** | **Control** | **Total** |
| --- | --- | --- | --- |
| **PrEP Experienced + High risk** |  |  |  |
| No. | 39 | 59 | 98 |
| % | 39.8 | 60.2 | 100 |
| **PrEP Naive + High risk** |  |  |  |
| No. | 91 | 137 | 228 |
| % | 39.9 | 60.1 | 100 |
| **PrEP Experienced + Low risk** |  |  |  |
| No. | 20 | 29 | 49 |
| % | 40.8 | 59.2 | 100 |
| **PrEP Naive + Low risk** |  |  |  |
| No. | 50 | 75 | 125 |
| % | 40.0 | 60.0 | 100 |

# Text B: List experiment method

The **double list experiment** introduces statistical noise in order to mask individual responses and so encourage truthful reporting [1]. Direct elicitation splits the participants into the treatment arm and the control arm, while the list experiment further splits each arm into a list experiment treatment arm (4 statements - 1 condom use, 3 non-sensitive statements) and a list experiment control arm (3 non-sensitive statements). The additional random assignment increases confidence intervals and reduces statistical power compared to direct elicitation.

In our experiment, we use a double list experiment (i.e. two lists for just 1 statement). The bracketed statement in bold italics is the statement of interest that we would like to measure the prevalence of. Respondents were split into two groups. Group A saw 4 statements in List 1 and 3 statements in List 2. Group B saw 3 statements in List 1 and 4 statements in List 2, i.e. all respondents had a chance to see the statement of interest.

This is the list experiment procedure used in the survey:

List 1

"I am going to read you four sentences. I ask you to count how many of these sentences you agree with. You do not have to tell me which sentences you agree with, just how many sentences you agree with.

To help you count the number of sentences you agree with, I am going to give you 3(**4)** marbles. Please place these marbles in your right hand and keep your hands behind your back. If you agree with the sentence I am reading, please transfer one marble from your right hand to your left hand. If you disagree with this sentence, do nothing. After all the sentences are read, you will tell me how many sentences you agree with. This number should match the number of marbles you have in your left hand. I will now read these sentences.

"

1. It is safer to take the client home than to a hotel.

***2. (I used a condom the last time I had sex with a client.)***

3. I prefer the client to pay me before the act.

4. Monday is the day I have the most clients.

Can you tell me how many of these three **(four)** sentences you agree with?

List 2

"I am going to read you four sentences. I ask you to count how many of these sentences you agree with. You do not have to tell me which sentences you agree with, just how many sentences you agree with.

To help you count the number of sentences you agree with, I am going to give you 3(**4)** marbles. Please place these marbles in your right hand and keep your hands behind your back. If you agree with the sentence I am reading, please transfer one marble from your right hand to your left hand. If you do not agree with this sentence, do nothing. Once all the sentences are read, you will tell me the number of sentences you agree with. This number should correspond to the number of marbles you have in your left hand. I will now read these sentences.

"

1. The majority of my clients are Senegalese

2. I used a condom the last time I had sex with a client.

3. I usually spend the whole night with the client

4. I most often solicit clients by phone

Can you tell me how many of these three **(four)** sentences you agree with?

With the list experiment, we are unable to tie a respondent’s response to the respondent itself. Instead, group level differences in the number of statements is used to infer the proportion of respondents agrees with the statement of interest. Therefore, considerable measurement noise arises from the presence of other statements compared to direct elicitation. When using it on the PrEP experiment, it is also necessary that the PrEP referral intervention or PrEP uptake did not affect the probability of agreeing with other statements.

For analysis of the list experiment within the PrEP experiment, we use only 4 statement responses from the respondents.

We first calculate the following quantities:

(1a) Prevalence of condom use with last client in List 1 for the 4 statements version

= Average no. of statements agreed with in the 4 statements version by PrEP treatment group

- Average no. of statements agreed with in the 4 statements version by PrEP control group

(1b) Prevalence of condom use with last client in List 1 for the 3 statements version

= Average no. of statements agreed with in the 3 statements version by PrEP treatment group

- Average no. of statements agreed with in the 3 statements version by PrEP control group

(2a) Prevalence of condom use with last client in List 2 for the 4 statements version

= Average no. of statements agreed with in the 4 statements version by PrEP treatment group

- Average no. of statements agreed with in the 4 statements version by PrEP control group

(2b) Prevalence of condom use with last client in List 2 for the 3 statements version

= Average no. of statements agreed with in the 3 statements version by PrEP treatment group

- Average no. of statements agreed with in the 3 statements version by PrEP control group

We then do something analogous to a difference-in-differences, i.e. we calculate [(1a) – (1b)] and [(2a) – (2b)]. This negates the differences in the non-sensitive statements between the PrEP treatment and the control group, essentially trying to mitigate the problem that PrEP referral intervention or PrEP uptake could have affected the probability of agreeing with other statements.

We then get a combined weighted average of the differences by weighting [(1a) – (1b)] and [(2a) – (2b)] by the number of respondents in the PrEP treatment group in each list who saw the 4 statements version. We use bootstrap with 1000 iterations to get standard errors for this quantity.

**References**

[1] Blair G, Imai K. Statistical analysis of list experiments. Political Analysis. 2012 Jan;20(1):47-77.

# Text C: Colorbox method

The **colorbox method** blinds the respondent’s response from the enumerator [1-2]. The respondent selects a sealed envelope in a bowl of sealed envelopes. A sealed envelope contains several coupons in a sealed envelope, each with a black or white box which corresponds to a “yes”/”no” response. Each coupon comes with a unique PIN code that is not reused in the survey. The respondent tears the PIN code of her response and hands it to the enumerator, thereby ensuring confidentiality of the response. Researchers have the mapping of the PIN code to the responses, while enumerators do not, thereby blinding the enumerators to the respondent’s answers, while researchers are able to recover individual responses to achieve precise estimates.

The colorbox section starts with an information phase where information is provided to the respondent about the method, a training phase where the respondent learns how to use the coupons to answer questions and an actual phase where a series of sensitive questions were asked as well as two verification questions so that we can check the respondent’s understanding (Are you a female? / Are you a male?)- one at the beginning and one near the end of the section. We drop observations that do not answer the verification questions correct in the analysis (19 respondents). As the colorbox method retrieves a respondent’s answer at the individual level, analysis steps are the same as direct elicitation.

Coupons in the training coupons are marked with a “T”. Coupons in the actual phase are the same but have no “T” marking. Examples of training coupons:


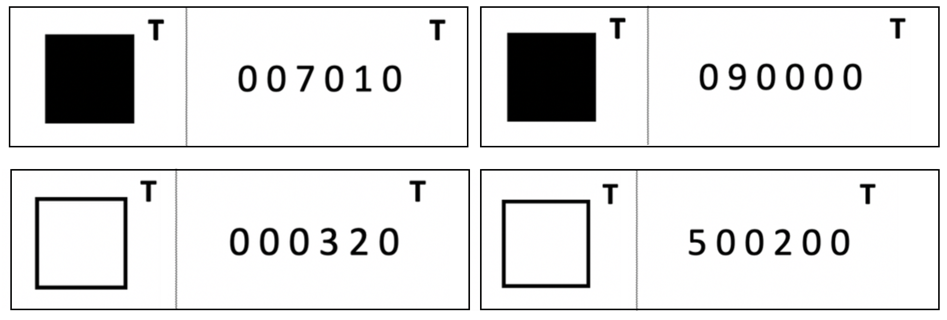


The visual aid mapping the box to “yes”/”no” shown to the respondent when answering the questions:


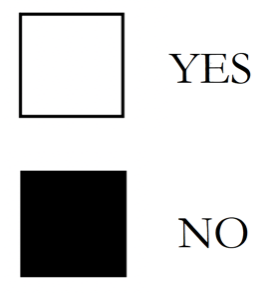


The colorbox procedure in the survey is as follows:

**INFORMATION PHASE**

READ: To protect your privacy, researchers designed this section to answer sensitive questions in complete confidentiality.

I will now ask you questions, but you will not give me the answers directly. Instead, you will give me a secret code.

I will not be able to know what your answer is because there are 1 million possible codes. Only people far away who analyze the data have the secret key to understand this code. Your answers will be protected by European legislation so that no one in Senegal can make the link between your answers and you.

**TRAINING PHASE**

READ: Before we start, we will first do a training together with the T envelope. Just for this test phase, we will place the test coupons on the table, to see if you understand the process.

I will ask you questions whose answer is always YES or NO. YES corresponds to the color white and NO to the color black. Instead of answering me YES or NO, I will ask you to take the coupon with the color that corresponds to the answer, tear it up and give me the code with the numbers.

You must be very careful when tearing the coupon, the secret code must be clearly readable and I must not see part of the color of the coupon otherwise I will know your answer.

"READ: We are going to do a test. If I ask you ""Are we in Senegal?""

"

Please use a new coupon for the next question.

DO NOT READ: Point to the white box on the visual aid when you say "yes" and point to the black box on the visual aid when you say "no". Repeat the question and the answer options twice slowly.

"READ: Now if I ask you: ""Are we in Mali?""

"

Please tear up the correct coupon and give me the 6-digit code. I'll enter this code into the system like this. And then I'll give it back to you like this.

READ: Please put away the training coupons. We won't be using them again.

**ACTUAL PHASE**

READ: Now we're going to start the real trick. Here's a bowl of envelopes marked A. These envelopes have been sealed by someone else. Each envelope contains different coupons. Each code only appears once in our study. You will use a new secret code for each new question.

Take a sealed envelope marked A. Don't show me its contents.

There are 9 questions in total. At the end of this session, you will leave with your envelope and codes, so no one will know what you answered.

"I will be turning around throughout this session to give you more privacy.

Are we ready to begin?"

READ: Now we'll start with the questions.

READ: The first question is about your gender.

"Are you a woman?

""Yes"" - ""white"", ""No"" - ""black"".

Please tear off the correct coupon and give me the 6-digit code.

INTERVIEWER: Enter the 6-digit code:"

Please use a new coupon for the next question.

READ: Please think about your LAST INTERCOURSE WITH A CLIENT

"Did you use a condom during sexual intercourse with your last client? If multiple intercourse, refer to the last intercourse.

""Yes"" - ""white"", ""No"" - ""black"".

Please tear off the correct coupon and give me the 6-digit code.

INTERVIEWER: Enter the 6-digit code:"

Please use a new coupon for the next question.

READ: Think again about this last client

"With this last client, did you have anal intercourse?

""Yes"" - ""white"", ""No"" - ""black"".

Please tear off the correct coupon and give me the 6-digit code.

INTERVIEWER: Enter the 6-digit code:"

READ: Please think about your NEXT-LAST INTERCOURSE WITH A CLIENT

"Did you use a condom during intercourse with your second-to-last client? If multiple intercourse, refer to the last intercourse

""Yes"" - ""white"", ""No"" - ""black"".

Please tear off the correct coupon and give me the 6-digit code.

INTERVIEWER: Enter the 6-digit code:"

Please use a new coupon for the next question.

READ: Think again about that second to last client

"With that second to last client, did you have anal intercourse?

"Yes" - "white", "No" - "black".

Please tear off the correct coupon and give me the 6-digit code.

INTERVIEWER: Enter the 6-digit code:"

Please use a new coupon for the next question.

READ: Please think about your THIRD last intercourse with a client, i.e. your LAST INTERCOURSE BEFORE THE PENULTIMATE INTERCOURSE WITH A CLIENT.

"Did you use a condom during sexual intercourse with your third last client? If multiple intercourse, refer to the last intercourse

""Yes"" - ""white"", ""No"" - ""black"".

Please tear off the correct coupon and give me the 6-digit code.

INTERVIEWER: Enter the 6-digit code:"

Please use a new coupon for the next question.

READ: Think about this client again

"With this third last client, did you have anal intercourse?

""Yes"" - ""white"", ""No"" - ""black"".

Please tear off the correct coupon and give me the 6-digit code.

INTERVIEWER: Enter the 6-digit code:"

Please use a new coupon for the next question.

READ: This question is about your sex again.

"Are you a boy?

""Yes"" - ""white"", ""No"" - ""black"".

Please tear off the correct coupon and give me the 6-digit code.

INTERVIEWER: Enter the 6-digit code:"

Please use a new coupon for the next question.

READ: This is the last question.

"Are you HIV positive?

""Yes"" - ""white"", ""No"" - ""black"".

Please tear off the correct coupon and give me the 6-digit code.

INTERVIEWER: Enter the 6-digit code:"

**References**

[1] Lépine A, Toh WQ, Treibich C. Colorbox: a novel method for eliciting sensitive behaviours in face-to-face interviewer-led surveys. Unpublished.

[2] Valente C, Toh WQ, Jalingo I, Lépine A, de Paula Á, Miller G. Are self-reported fertility preferences biased? Evidence from indirect elicitation methods. Proceedings of the National Academy of Sciences. 2024 Aug 20;121(34):e2407629121.

# Table A: Survey questions used to derive outcomes

|  | **Survey question (translated)** | **Original survey question** |
| --- | --- | --- |
| **PrEP use** | Are you currently on PrEP to prevent HIV? | Êtes-vous actuellement sous PrEP pour le but de prévenir le VIH ? |
| **Ever used PrEP in past year** | Have you started taking PrEP this year or last year? | Avez-vous déjà commencé à prendre de PrEP cette année ou l'année dernière? |
| **Condom used with last/penultimate/third last client** | Did you use a condom during sexual intercourse with your last/penultimate/third last client? If multiple intercourses, refer to the last intercourse. | Avez-vous utilisé un préservatif lors du rapport sexuel avec ce client ? Si plusieurs rapports, se référer au dernier rapport. |
| **Self-reported sexual risk-taking** | When it comes to your attitude towards risk, how would you rate yourself on a scale from 0 to 10. 0 is for people who are very cautious and try to limit the risks in life. 10 is for people who are more attracted to adventure and like to take risks.  ... regarding your sexual behavior? | En matière d'attitude à l'égard du risque, comment vous placez-vous sur une échelle allant de 0 à 10. 0 correspond aux personnes très prudentes qui s’efforcent de limiter les risques de l’existence. 10 correspond aux personnes les plus attirées par l’aventure qui aiment prendre des risques.  ... en ce qui concerne vos comportements sexuels ? |
| **Clients in a typical week** | In a typical week, how many different clients did you have? | Au cours une semaine typique, combien de clients différents avez-vous eu ? |
| **Average sex acts per client** | [Question posed with respect to the last 3 clients] How many times have you had sex with this client? | Combien de rapports sexuels avez-vous eu avec ce client ? |
| **Perceived HIV risk of client** | On a scale of 0 to 10, in your opinion, was this client a person at risk for AIDS? 0 corresponds to no risk of AIDS, 10 to a very high risk of AIDS. | Sur une échelle allant de 0 à 10 selon vous ce client était-il une personne à risque pour le Sida ? 0 correspond à aucun risque Sida, 10 à un très grand risque Sida |
| **Share of regular clients in a typical week** | |  |
| Casual clients | Typically, how many different casual customers do you have in a week? | En général, combien de clients occasionnels différents avez-vous en une semaine ? |
| Regular clients | Typically, how many different regular customers do you have in a week? | En général, combien de clients réguliers différents avez-vous en une semaine ? |
| **Oral sex with last/penultimate/third last client** | Did you give this customer oral sex? | Avez-vous fait une fellation à ce client ? |
| **HIV/STI risk perceptions (all participants)** | |  |
| HIV risk without condom | If you now have UNPROTECTED SEX with someone who is HIV-positive, what is the likelihood that you will also become infected as a result of that sexual encounter? | Si vous avez maintenant un RAPPORT NON PROTÉGÉ avec une personne séropositive, quelle est la probabilité que vous soyez infectée vous aussi à la suite de ce rapport ? |
| HIV risk with condom | If you now have PROTECTED SEX with someone who is HIV-positive, what is the likelihood that you will also become infected as a result of that sexual encounter? | Si vous avez maintenant un RAPPORT PROTÉGÉ avec une personne séropositive, quelle est la probabilité que vous soyez infectée vous aussi à la suite de ce rapport ? |
| STI risk without condom | If you now have UNPROTECTED SEX with someone who has an STI other than HIV, what is the likelihood that you will become infected with that STI as a result of that sexual encounter? | Si vous avez maintenant un RAPPORT NON PROTÉGÉ avec une personne qui a une IST autre que le VIH, quelle est la probabilité que vous soyez infecté par cette IST à la suite de ce rapport ? |
| STI risk with condom | If you now have PROTECTED SEX with someone who has an STI other than HIV, what is the likelihood that you will become infected with that STI as a result of that sexual encounter? | Si vous avez maintenant un RAPPORT PROTÉGÉ avec une personne qui a une IST autre que le VIH, quelle est la probabilité que vous soyez infecté par cette IST à la suite de ce rapport ? |
| **HIV risk perceptions [PrEP users only]** | |  |
| Without condom or PrEP | If you are not using any protection, that is, if you 1) have UNPROTECTED sex with an HIV-positive person, and 2) STOP taking PrEP, what is the likelihood that you will become infected with HIV as a result of that sexual encounter? | Si vous n'utilisez aucune protection, autrement dit, si vous 1) avez un rapport NON PROTEGE avec une personne séropositive, et 2) ARRÊTEZ de prendre la PrEP, quelle est la probabilité que vous soyez infectée par le virus du VIH à la suite de ce rapport? |
| Without condom, with PrEP | If you 1) have UNPROTECTED sex with an HIV-positive person, and 2) CONSISTENTLY TAKE PrEP, what is the likelihood that you will become infected with HIV as a result of that sexual encounter? | Si vous 1) avez un rapport NON PROTEGE avec une personne séropositive, et 2) PRENEZ la PrEP de façon CONSISTANTE, quelle est la probabilité que vous soyez infectée par le virus du VIH à la suite de ce rapport ? |
| With condom, without PrEP | If you 1) have PROTECTED sex with an HIV-positive person, and 2) STOP taking PrEP, what is the likelihood that you will become infected with HIV as a result of that sexual encounter? | Si vous 1) avez un rapport PROTEGE avec une personne séropositive, et 2) ARRÊTEZ de prendre la PrEP, quelle est la probabilité que vous soyez infectée par le virus du VIH à la suite de ce rapport? |
| With condom & PrEP | If you 1) have PROTECTED sex with an HIV-positive person, and 2) TAKE PrEP CONSISTENTLY, what is the likelihood that you will become infected with the HIV virus as a result of that sexual encounter? | Si vous 1) avez un rapport PROTEGE avec une personne séropositive, et 2) PRENEZ la PrEP de façon CONSISTANTE, quelle est la probabilité que vous soyez infectée par le virus du VIH à la suite de ce rapport ? |

*Notes*. PrEP, Pre-Exposure Prophylaxis.

# Table B: Pre-attrition cohort characteristics by treatment status, at baseline (2020)

|  | **Control (C)**  **(n=200)** | **Treatment (T) (n=300)** |
| --- | --- | --- |
|  |  |  |
| **Sociodemographics** |  |  |
| Age (years) | 38.5 (9.7) | 38.9 (9.7) |
| Ever went to school |  |  |
| Yes | 100 (50%) | 143 (48%) |
| No | 100 (50%) | 157 (52%) |
| Marital status |  |  |
| Never married | 41 (20%) | 66 (22%) |
| Married | 2 (1%) | 2 (1%) |
| Divorced/Separated/  Widowed | 157 (78%) | 232 (77%) |
| Household is indebted |  |  |
| Yes | 106 (54%) | 166 (56%) |
| No | 91 (46%) | 132 (44%) |
| **Sex work** |  |  |
| Registered with authorities |  |  |
| Yes | 93 (47%) | 136 (45%) |
| No | 107 (53%) | 164 (55%) |
| Clients in typical week (No.) | 6.2 (5.2) | 6.6 (6.1) |
| Clients in last 7 days (No.) | 2.3 (3.7) | 2.7 (4.4) |
| Sex work income  in last 7 days ('000 CFAF) | 23.1 (53.7) | 22.4 (53.1) |
| average monthly ('000 CFAF) | 137.8 (126.4) | 123 (100.1) |
| share of total income (%) | 87 (20) | 82 (23) |
| Non-sex work income, average monthly ('000 CFAF) | 20.7 (37.1) | 30.7 (62.0) |
| Sexual risk-taking (0=Limits risks, 10=Likes to take risk) |  |  |
| Low (<=1) | 111 (56%) | 171 (57%) |
| High (> 1) | 89 (44%) | 129 (43%) |
| Condom use with last client |  |  |
| Yes | 193 (97%) | 292 (98%) |
| No | 6 (3%) | 7 (2%) |
| Share of regular clients in typical week (%) | 72 (29) | 71 (30) |

*Notes: Table shows characteristics of the cohort randomized to treatment and control arms prior to attrition from the 2022 endline survey. Table shows mean (SD) for continuous/integer variables and n (%) for binary/categorical variables. See SM Text A for definitions of randomization strata. CFAF refers to CFA franc.*

# Table C: Probability of analysis cohort inclusion by treatment assignment and baseline (2020) characteristics (n=500)

|  | **Coefficient** | **95% CI** | ***p*** |
| --- | --- | --- | --- |
| **Model 1** |  |  |  |
| Treatment arm (0, 1) (pp) | -2.3 | [-11.0 , 6.3] | 0.600 |
|  |  |  |  |
| **Model 2** |  |  |  |
| Treatment arm (0, 1) (pp) | -2.8 | [-12.0 , 6.5] | 0.557 |
|  |  |  |  |
| Age (years) | 0.000 | [-0.006 , 0.005] | 0.893 |
| No School (0, 1) (pp) | 5.0 | [-4.0 , 14.1] | 0.276 |
| Marital status (ref: Separated/Widowed) |  |  |  |
| Never married (0, 1) (pp) | -0.003 | [-12.4 , 11.8] | 0.958 |
| Married (0, 1) (pp) | 0.209 | [-14.4 , 56.2] | 0.246 |
| Household indebted (0, 1) (pp) | 0.112 | [2.0 , 20.5] | 0.017 |
| Registered sex worker (0, 1) (pp) | -0.035 | [-13.7 , 6.7] | 0.507 |
| Clients in typical week (No.) | 0.007 | [-0.005 , 0.018] | 0.254 |
| Clients in last 7 days (No.) | -0.001 | [-0.021 , 0.019] | 0.944 |
| Sex work income in last 7 days ('000 CFAF) | 0.000 | [-0.001 , 0.002] | 0.551 |
| Average monthly sex work income ('000 CFAF) | 0.000 | [-0.001 , 0.000] | 0.144 |
| Average monthly non-sex work income ('000 CFAF) | 0.000 | [-0.002 , 0.001] | 0.838 |
| Share of sex work income in total income (0, 1) (pp) | 8.7 | [-24.4 , 41.8] | 0.606 |
| Sexual risk-taking | -0.035 | [-0.137 , 0.067] | 0.507 |
| Randomisation strata (ref: PrEP never used + Low risk-taking) |  |  |  |
| PrEP ever used + High risk-taking (0, 1) (pp) | 18.5 | [3.2 , 33.9] | 0.018 |
| PrEP never used + High risk-taking (0, 1) (pp) | 3.2 | [-10.4 , 16.7] | 0.647 |
| PrEP ever used + Low risk-taking (0, 1) (pp) | 7.9 | [-4.3 , 20.1] | 0.206 |
| Self-reported sexual risk-taking |  |  |  |
| (0=Limits risks, 10=Likes to take risk) (Ref: >0) |  |  |  |
| Low (<=1) (0, 1) (pp) | 11.2 | [-0.9 , 23.3] | 0.069 |
|  |  |  |  |
| Used condom with last client (0, 1) (pp) | -8.9 | [-37.4 , 19.6] | 0.542 |
| Proportion of regular clients in typical week (0-1) (pp) | 2.9 | [-14.2 , 20.0] | 0.743 |

*Notes: Logistic regression estimates of change in probability of analysis cohort inclusion with treatment assignment and baseline covariates. Binary regressors are indicated using (0, 1), while (0-1) refers to regressors whose range is capped between 0 and 1. For both of these regressors, the coefficients are risk differences and expressed in percentage points (pp). Model 1 includes only the treatment arm variable as a regressor. Model 2 includes the treatment arm variable and the baseline covariates shown. Randomisation strata are defined in SM Text A. PrEP, Pre-Exposure Prophylaxis; p, p-value; CFAF, CFAF franc.*

# Table D: Characteristics of analysis cohort in 2020 and at endline (2022) (n=308)

|  | **2020** | **2022** |
| --- | --- | --- |
| **Sociodemographics** |  |  |
| Age (years) | 38.9 (9.4) | 41.2 (9.2) |
| Ever went to school |  |  |
| Yes | 141 (46%) | 167 (54%) |
| No | 167 (54%) | 141 (46%) |
| Marital status |  |  |
| Never married | 63 (20%) | 37 (12%) |
| Married | 3 (1%) | 3 (1%) |
| Divorced/Separated/Widowed | 242 (79%) | 268 (87%) |
| Household is indebted |  |  |
| Yes | 179 (59%) | 182 (59%) |
| No | 124 (41%) | 126 (41%) |
|  |  |  |
| **Sex work** |  |  |
| Registered |  |  |
| Yes | 138 (45%) | 135 (44%) |
| No | 170 (55%) | 173 (56%) |
| Clients in typical week (No.) | 6.5 (6.0) | 6.8 (6.7) |
| Clients in last 7 days (No.) | 2.6 (4.1) | 2.7 (5.2) |
| Sex work income  in last 7 days ('000 CFAF) | 23.1 (52.2) | 19.4 (33) |
| average monthly ('000 CFAF) | 124.5 (105.2) | 138.6 (127.9) |
| share of total income (%) | 84 (21) | 85 (21) |
| Non-sex work income, average monthly ('000 CFAF) | 24.8 (48.8) | 25.6 (57.6) |
| Sexual risk-taking (0=Limits risks, 10=Likes to take risk) | |  |
| Low (<=1) | 140 (45%) | 166 (54%) |
| High (>1) | 168 (55%) | 142 (46%) |
| Condom used with last client |  |  |
| Yes | 298 (97%) | 269 (87%) |
| No | 9 (3%) | 39 (13%) |
| Share of regular clients in typical week (%) | 72 (30) | 52 (22) |

*Notes: Table shows mean (SD) for continuous/integer variables and n (%) for binary/categorical variables. CFAF refers to CFA franc.*

# Table E: Outcome means for control and treatment groups at endline

|  | **Control (n = 126)** | **Treatment (n = 182)** | |  |
| --- | --- | --- | --- | --- |
| **Condom used** (Yes / No) |  |  |  | |
| With last client (%) | 84.9 | 89.0 |  | |
| With all last 3 clients (%) | 67.5 | 79.1 |  | |
| **HIV risk perception** (Yes / No) | | | | |
| High risk without condom (%) | 82.4 | 86.2 |  | |
| Low risk with condom (%) | 72.0 | 75.7 |  | |
| High risk without & low risk with condom (%) | 59.2 | 65.7 |  | |
| **STI risk perception** (Yes / No) | | | | |
| High risk without condom (%) | 81.0 | 80.8 |  | |
| Low risk with condom (%) | 69.0 | 72.5 |  | |
| High risk without & low risk with condom (%) | 55.6 | 58.6 |  | |
| **Secondary outcomes** |  |  |  | |
| **Binary outcomes** |  |  |  | |
| High sexual risk- taking (%) | 45.2 | 46.7 |  | |
| High HIV risk of last 3 clients (%) | 37.3 | 49.5 |  | |
| Oral sex with ≥ 1 of last 3 clients (%) | 30.2 | 22.0 |  | |
| **Non-binary outcomes** |  |  |  | |
| Clients in typical week (No.) | 6.523 | 7.005 |  | |
| Average sex acts per client (No.) | 1.207 | 1.168 |  | |
| Share of regular clients in typical | 53.7 | 51.0 |  | |
| week (%) |  |  |  | |

*Notes: STI, sexually-transmitted infections.*

# Table F: Mean predicted outcomes under counterfactual of no PrEP use

|  |  | | |
| --- | --- | --- | --- |
|  |  | **No *PrEP use*** | **Not *Ever used PrEP in past year*** |
| **Condom used (Yes / No)** |  |  |  |
| With last client (%) |  | 85.6 | 88.3 |
| With all last 3 clients (%) |  | 71.1 | 75.0 |
| **HIV risk perception (Yes / No)** | | | |
| High risk without condom (%) |  | 83.5 | 84.2 |
| Low risk with condom (%) |  | 82.5 | 82.5 |
| High risk without & low risk with condom (%) |  | 69.1 | 70.0 |
| **STI risk perception** | | | |
| High risk without condom (%) |  | 77.3 | 77.5 |
| Low risk with condom (%) |  | 74.2 | 74.2 |
| High risk without & low risk with condom (%) |  | 55.7 | 56.7 |

*Notes: PrEP, Pre-Exposure Prophylaxis, STI, sexually-transmitted infections. Adjusted risk differences (RDs) in binary outcomes by each measure of PrEP use were estimated using a recursive bivariate probit model.* *We also calculated each estimated RD relative to the mean counterfactual outcome if there were no PrEP use. The mean predicted counterfactual outcome was obtained by averaging over PrEP users in the bivariate probit, i.e. the estimand is average treatment effect of the treated. The counterfactual mean differs for PrEP use and Ever used PrEP in past year because there is a different bivariate probit model in each case and the target populations are different (PrEP users vs. users who ever used PrEP in past year).*

# Table G: Unadjusted risk differences (RDs) in condom use by PrEP referral and use

|  | **PrEP referral (n = 308)** | | | **PrEP use (n = 308)** | | | **Ever used PrEP in past year (n = 308)** | | |
| --- | --- | --- | --- | --- | --- | --- | --- | --- | --- |
|  | **RD** | **95% CI** | ***p*** | **RD** | **95% CI** | ***p*** | **RD** | **95% CI** | ***p*** |
| **Condom used (Yes / No)** |  |  |  |  |  |  |  |  |  |
| With last client (pp) | 4.1 | [-3.6, 11.8] | 0.299 | 10.1 | [-8.3, 28.4] | 0.281 | 9.4 | [-7.4, 26.2] | 0.275 |
|  |  |  |  |  |  |  |  |  |  |
| With all last 3 clients (pp) | 11.7 | [1.6, 21.8] | 0.024 | 28.0 | [7.7, 48.3] | 0.007 | 25.9 | [7.6, 44.1] | 0.006 |

*Notes: PrEP refers to pre-exposure prophylaxis. RD, CI and p refer to risk differences, confidence interval and p-value respectively. pp refers to percentage points. As top panel of Table 2 but without adjustment for covariates. Risk differences (RD) caused by PrEP referral estimated by logistic regression. RD caused by PrEP use estimated by recursive bivariate probit with PrEP use instrumented with random assignment to PrEP referral. PrEP use is use at the endline survey. Ever used PrEP in past year is at the endline survey or any time in the preceding 12 months.*

# Table H: Adjusted risk differences in condom use by PrEP referral and use – robustness to reweighting analysis cohort and treatment group

|  |  | **PrEP referral (n = 308)** | | | **PrEP use (n = 308)** | | | **Ever used PrEP in past year (n = 308)** | | |
| --- | --- | --- | --- | --- | --- | --- | --- | --- | --- | --- |
|  |  | **RD** | **95% CI** | ***p*** | **RD** | **95% CI** | ***p*** | **RD** | **95% CI** | ***p*** |
| **Without reweighting (as Table 3)** | | | | | | | | | | |
| Condom used with last client (pp) |  | 3.3 | [-4.0, 10.6] | 0.376 | 07.9 | [-10.4, 26.3] | 0.397 | 7.4 | [-9.3, 24.0] | 0.386 |
| Condom used with all last 3 clients (pp) |  | 11.0 | [0.8, 21.2] | 0.034 | 25.8 | [5.2, 46.4] | 0.014 | 24.3 | [5.6, 42.9] | 0.011 |
| **Reweight analysis cohort to match pre-attrition cohort** | | | | | | | | | | |
| Condom used with last client (pp) |  | 7.3 | [-2.1, 16.7] | 0.128 | 15.7 | [-4.9, 36.2] | 0.137 | 14.7 | [-4.0, 33.3] | 0.123 |
| Condom used with all last 3 clients (pp) |  | 12.2 | [0.6, 23.8] | 0.039 | 25.9 | [3.9, 47.9] | 0.021 | 24.9 | [4.7, 45.1] | 0.016 |
| **Reweight treatment group to match control group in analysis cohort** | | | | | | | | | | |
| Condom used with last client (pp) |  | 3.4 | [-4.1, 11.0] | 0.375 | 8.7 | [-9.5, 27.0] | 0.349 | 8.1 | [-8.5, 24.6] | 0.201 |
| Condom used with all last 3 clients (pp) |  | 10.7 | [-0.0, 21.5] | 0.050 | 27.0 | [5.5, 48.5] | 0.014 | 24.9 | [5.6, 44.1] | 0.012 |

*Notes: PrEP refers to pre-exposure prophylaxis. RD, CI and p refer to risk differences, confidence interval and p-value respectively. pp refers to percentage points. Without reweighting are estimates from top panel of Table 3. Reweight analysis cohort to pre-attrition cohort* *gives estimates with entropy balancing (Hainmueller, 2012) weights used to match the analysis cohort (n=308) on moments of covariates of pre-attrition cohort (n = 500). Covariates are 2020 values of age, education, marital status, household indebtedness, sex worker registration status, number of clients, sex work revenue, non-sex work revenue, share of regular clients, self-reported condom use, self-reported sexual risk taking and previous PrEP experience. Reweight treatment group to match control group in analysis cohort gives estimates with entropy balancing weights to match the treatment group to moments of covariates of the control group in the analysis cohort. Covariates are the same, plus number of days in the 7 preceding the endline interview within Ramadan. The weights were generated using R’s ebal function. After reweighting, risk differences (RD) were estimated by regression. RD caused by PrEP referral estimated by adjusted logistic regression. RD caused by PrEP use were estimated by recursive bivariate probit with PrEP use instrumented with random assignment to PrEP referral. Adjustment for age, number of days in 7 preceding endline interview within Ramadan and 2020 values of marital status, FSW registration, self-reported sexual risk taking, prior PrEP experience and the (lagged) outcome.*

**References**

Hainmueller J. Entropy balancing for causal effects: A multivariate reweighting method to produce balanced samples in observational studies. Political analysis. 2012 Jan;20(1):25-46.

# Table I: Adjusted risk differences in condom use by PrEP referral and use – alternative measurements of condom use

|  | **Control group mean**  **(%)** | **PrEP referral (n = 308)** | | | **PrEP use (n = 308)** | | **Ever used PrEP in past year (n = 308)** | | | |  |
| --- | --- | --- | --- | --- | --- | --- | --- | --- | --- | --- | --- |
|  |  | **RD**  **(pp)** | **95% CI**  **(pp)** | **p** | **RD**  **(pp)** | **95% CI**  **(pp)** | **p** | **RD**  **(pp)** | **95% CI**  **(pp)** | **p** | |
| **Direct elicitation** |  |  |  |  |  |  |  |  |  |  | |
| Condom used with last client | 84.9 | 3.3 | [-4.0, 10.6] | 0.376 | 7.9 | [-10.4, 26.3] | 0.397 | 7.4 | [-9.3, 24.0] | 0.386 | |
|  |  |  |  |  |  |  |  |  |  |  | |
| *Condom used with penultimate client | 87.3 | 2.7 | [-4.2, 9.7] | 0.442 | 8.3 | [-5.5, 22.0] | 0.221 | 6.6 | [-4.7, 17.8] | 0.252 | |
| Condom used with all last 3 clients | 67.5 | 11.0 | [0.8, 21.2] | 0.034 | 25.8 | [5.2, 46.4] | 0.014 | 24.3 | [5.6, 42.9] | 0.011 | |
| **Colorbox elicitation** |  |  |  |  |  |  |  |  |  |  | |
| *Condom used with last client | 83.8 | 2.6 | [-4.7, 10.0] | 0.482 | 9.1 | [-15.0, 33.3] | 0.459 | 7.7 | [-10.8, 26.3] | 0.415 | |
| *Condom used with penultimate client | 88.1 | 2.8 | [-3.5, 9.2] | 0.379 | 9.3 | [-9.1, 27.8] | 0.321 | 7.0 | [-9.8, 23.8] | 0.413 | |
| Condom used with all last 3 clients | 73.5 | 4.2 | [-5.7, 14.2] | 0.405 | 12.0 | [-16.9, 40.9] | 0.416 | 11.1 | [-12.1, 34.4] | 0.347 | |
| **Double list experiment** |  |  |  |  |  |  |  |  |  |  | |
| *Condom used with last client | 69.4 | 1.1 | [-2.5, 6.0] | 0.548 | - | - | - | - | - | - | |

*Notes: PrEP refers to pre-exposure prophylaxis. RD, CI and p refer to risk differences, confidence interval and p-value respectively. pp refers to percentage points. Risk difference (RD) caused by PrEP referral estimated by adjusted logistic regression. RD caused by PrEP use estimated by recursive bivariate probit with PrEP use instrumented with random assignment to PrEP referral. Adjustment for age, indicator of number of days in last 7 within Ramadan during the 2022 survey interview, marital status in 2020, FSW registration, self-reported sexual risk taking in 2020, prior PrEP experience and the 2020 value of the outcome.*

** indicate outcomes specified in the study protocol and not included in* ***Table 3****. The double list experiment and the colorbox methods of indirectly eliciting condom use explained in* ***SM Text B*** *and* ***Text C****, respectively. Double list experiment used to elicit condom use only with the last client. This method does not measure condom use at the individual participant level, and so could not be used to estimate effects of PrEP use on condom use. This method used two lists. The first list gave a PrEP referral estimate of -18.5% (95% CI [-46.7%, 10.0%]), while the second list gave an estimate of -4.0% (95% CI [-27.2%, 18.0%]). This method embedded an elicitation experiment within the PrEP referral experiment, which added sampling variability and likely left the analysis underpowered. We report the estimate obtained with this method because it was specified in the study protocol. But it should be interpreted cautiously. The mean ITT difference implied by each of the two lists also hugely differs, although their confidence intervals overlap considerably because the confidence intervals are large. List 1: -14.5% (95% CI [-53.0%, 24.0%]); List 2: 14.5% (95% CI [-24.8%, 53.0%]).*

# Table J: Adjusted mean differences (MDs) in secondary outcomes and HIV/STI risk perceptions by PrEP referral and use – alternative specifications

|  |  | **PrEP referral (n = 308)** | | | **PrEP use (n = 308)** | | | **Ever used PrEP in past year (n = 308)** | | | | |
| --- | --- | --- | --- | --- | --- | --- | --- | --- | --- | --- | --- | --- |
|  |  | **MD** | **95% CI** | ***p*** | **MD** | **95% CI** | ***p*** | **MD** | **95% CI** | | ***p*** | |
|  | | | | | | | | | |  | |  |
| Self-reported sexual risk taking (0: Never, 10: Always) |  | -0.080 | [-0.526, 0.367] | 0.726 | -0.234 | [-1.548, 1.08] | 0.727 | -0.207 | [-1.368, 0.954] | | 0.727 | |
| Perceived HIV risk of last three clients (0: No risk, 10: Very high risk) |  | 0.198 | [-0.261, 0.656] | 0.398 | 0.583 | [-0.798, 1.963] | 0.409 | 0.515 | [-0.693, 1.722] | | 0.404 | |
| **HIV risk perceptions** | | | | | | | | | | | | |
| Without condom (1: Very unlikely, 5: Very likely) | 4.704 | 0.096 | [-0.085, 0.276] | 0.299 | 0.284 | [-0.259, 0.827] | 0.306 | 0.251 | [-0.231, 0.733] | | 0.308 | |
| With condom (1: Very unlikely, 5: Very likely) | 1.424 | -0.173 | [-0.364, 0.018] | 0.077 | -0.513 | [-1.084, 0.059] | 0.080 | -0.453 | [-0.953, 0.047] | | 0.077 | |
| Risk reduction: Without – With (-4, +4) | 3.280 | 0.270 | [-0.007, 0.547] | 0.057 | 0.801 | [-0.040, 1.641] | 0.063 | 0.707 | [-0.033, 1.448] | | 0.062 | |
| **STI risk perceptions** | | | | | | | | | | | | |
| Without condom (1: Very unlikely, 5: Very likely) | 4.690 | 0.005 | [-0.193, 0.202] | 0.964 | 0.013 | [-0.566, 0.592] | 0.964 | 0.012 | [-0.502, 0.525] | | 0.964 | |
| With condom (1: Very unlikely, 5: Very likely) | 1.556 | -0.112 | [1.763, -1.987] | 0.340 | -0.328 | [-1.001, 0.345] | 0.340 | -0.291 | [-0.887, 0.306] | | 0.340 | |
| Risk reduction: Without – With (-4, +4) | 3.135 | 0.119 | [-0.192, 0.431] | 0.454 | 0.348 | [-0.563, 1.259] | 0.454 | 0.308 | [-0.504, 1.121] | | 0.457 | |

*Notes: PrEP, pre-exposure prophylaxis; STI, sexually transmitted infections; MD, mean difference; CI, confidence interval; p, p-value for test of MD = 0. This table shows estimated mean differences (MDs) in outcomes derived from measures reported on 5-point or 11-point Likert scales. Tables 3 and 4 show estimated risk differences in binary outcomes derived from these measures. Here, the scales were interpreted as linear (1-5 or 1-10). MDs in these measures by PrEP referral and use were estimated by adjusted ordinary least squares and two-stage least squares (use instrumented by referral), respectively. Otherwise, notes to Tables 3 and 4 apply.*
