# Supplementary material for: Effect of pre-exposure prophylaxis on risky sexual behaviour of female sex workers in Dakar, Senegal: A randomised controlled trial
Source: PLoS Med. 2025 Aug 18;22(8):e1004458. doi: 10.1371/journal.pmed.1004458 (PMC12407539; doi:10.1371/journal.pmed.1004458)
Supplement: Attachment — Submitted filename: PrEP - PlosMed comments-3.pdf [file pmed.1004458.s008.pdf]

We thank the editor and reviewers for spending so much time and effort reading the paper carefully and making detailed, insightful comments that have been tremendously helpful. This document explains the changes made in response to each comment. For the sake of brevity and clarity, we adopt a matter-of-fact style, without repeating thanks for individual comments. Please do not interpret this as a lack of gratitude for really great comments.

We numbered the comments to facilitate cross-referencing. Comments are in bold. Responses are in plain text. Quotes from the manuscript are in italics.

Editor

1. The topic is both important and timely, also in light of a recent systematic review (Murchu et al., 2022) that highlighted the paucity of data on PreP-associated risk compensation.

Response. We cite this review in the first paragraph of the paper and have added the following sentence at the start of the second paragraph.

> *"There is a paucity of evidence on risk compensatory behaviour in response to PrEP. [2]"*

We also cite the review in the new first paragraph of the Discussion.

2. The study appears to be at high risk of bias, and the advance over existing knowledge with clear implications for patient care, public policy, or clinical research agendas needs to be made much clearer.

Response.

Bias. We have taken further steps to assess and reduce the risk of bias.

First, we used entropy balancing to reweight the (post-attrition) analysis sample to match the pre-attrition sample on moments (not just means) of covariates (Reviewer 1, Comment 3). Our main finding of no negative effect of PrEP on condom use is robust to this analysis (SM Table S6), which, as with robustness to covariate adjustment, suggests it is unlikely that the finding is due to bias from selective attrition.

Second, we confirmed that the main finding is also robust to using entropy balancing to match the treatment group to the control group in the analysis sample (Reviewer 1, Comment 4, SM Table S6), which is consistent with no substantial bias from selective non-compliance..

Third, we added an ex-post power calculation to show the extent to which greater than expected attrition and non-compliance reduced power (Reviewer 3, Comment 17).

Fourth, we confirmed that the finding of no negative PrEP effect on self-reported condom use is robust to using two indirect elicitation methods that are designed to reduce reporting errors (Reviewer 1, Comment 2). This, along with no statistically significant effect on secondary outcomes, is consistent with the finding reflecting a true lack of risk compensation in response to PrEP and not merely arising from social desirability bias that induced participants to overreport condom use.

In addition to these new analyses, the initial submission already contained analyses intended to assess and reduce the risk of bias. Specifically, we showed that the treatment and control groups in the (post-attrition) analysis sample are well balanced on covariates and we used regression to adjust for covariates. Also, we corrected estimates of effects of PrEP use for non-compliance by using randomisation to PrEP referral to instrument PrEP use. This ensured that these effects were identified only from the (trial-induced) random variation in PrEP.

While a number of (further) steps were taken to reduce risks of bias, we edited the text to further acknowledge the potential for bias. For example:

Abstract

*"**Interpretation** This study did not find evidence that PrEP referral or PrEP use increased self-reported risky sex behaviour of FSWs in one location, at least in the short-term. The robustness of this finding should be tested with additional data collected in other settings from larger samples, with longer follow-up and using survey instruments that allow further examination of whether PrEP users are more likely to overreport condom use."*

Author summary

"*The evidence from this study, which was based on a moderately sized sample in one location, … this finding could change if the belief that PrEP is more effective when combined with condom use were to weaken, and robustness of the result should be tested in other settings, with larger samples and longer follow-up.*"

Limitations

"This study had several limitations. First, … Second, … Third, … Fourth, … Fifth, …. Sixth, … Seventh, … ."

We have made considerable effort to reduce the risk of bias and acknowledge the risk that exists. It is worth keeping in mind that the risk partly arises from study of a marginalised population of female sex workers (FSWs) who, without registration, are engaged in an illegal

economic activity. Longitudinal study of this population is particularly challenging given many FSWs have no fixed address, women move in and out of the occupation, and they switch between unregistered and registered status. The vulnerability of FSWs, the stigma attached to their occupation and the sensitivity of sex-related outcomes add to the research challenge. Winning the trust of the study participants is critical.

The study built on seven years of research with a cohort of FSWs' and peer leaders. This long-term relationship provided an invaluable opportunity to estimate the effect of PrEP on sexual behaviour. While we acknowledge the risk that attrition, non-compliance, low power and reporting errors could potentially introduce bias, it is important to be alert to the other risk that not studying marginalised populations, such as FSWs, leaves policymakers without any evidence to guide the design of policies and programmes that affect these populations..

Advance in knowledge. We have added to the Discussion:

> *"This study contributed to sparse evidence on whether the important health benefits PrEP achieves through reduction in the risk of HIV are partially offset by risk compensatory behaviour that increases risks of STI that PrEP does not protect against [2]. To our knowledge, it is the first randomised experiment in Africa to have estimated effects of PrEP on behaviour of female sex workers (FSWs), a high-risk group."*

Implications. We have edited and extended the implications part of the Author Summary to read as follows:

> *"It is generally believed that prescribing PrEP to populations at high risk of HIV infection improves HIV prevention, despite some offsetting risk compensation, but may have a negative impact on the control of other sexually transmitted infections (STI). The evidence from this study, … does not lend support to concerns about a negative spillover from PrEP to more risky sex behaviour of FSWs. While this is a positive result with respect to the potential for PrEP to deliver important net health benefits by reducing HIV risk without inducing substantial offsetting increases in STI risk, …"*

In the Limitations sub-section, we have identified specific evidence gaps that remain after this study and should be addressed in future research in other settings undertaken with larger samples, longer follow-up and with further examination of reporting errors in sex behaviour.

3. The self-reported outcome measures on condom use are known to be prone to social desirability bias. There were no biological outcomes related to condom use, such as STIs or vaginal detection of prostate-specific antigen or Y-chromosomal DNA,

which have been used in previous studies (see e.g. Giguère et al. 2019). This probably cannot be remedied, but should be discussed in greater detail.

Response. In the Limitations sub-section, we added:

> *"Future research should further investigate whether PrEP programmes influence incentives to misreport condom use. In this study, without biomarker outcomes, such as STI diagnoses or the detection of prostate-specific antigen (PSA) and Y-chromosomal DNA, it is not possible to test whether the finding that PrEP did not increase unprotected intercourse is robust to using more objective measures of that outcome. While PSA and Y-chromosomal DNA have been used to measure unprotected sex, these methods also have limitations, such as short detection windows, potential discrepancies between markers [29] and complications like condom slippage and differentiating between sex with clients and boyfriends. Logistical, ethical and participant comfort considerations made it impractical to use biomarkers in this study setting."*

Please see our response to the previous comment at *Bias*, **Limitations**

4. The apparent change in the outcome measure pointed out by the careful statistical reviewer will be important to address.

Response. Please see our response to Reviewer 1, Comment 2.

5. The results of this study seem to be on the inconclusive side, which is only partially acknowledged by the authors.

Response. As documented in response to Comment 2, we have edited and extended the Limitations sub-section substantially to acknowledge study limitations, including limited power. We have stated caveats on the study conclusions that follow from the limitations. We have edited the final paragraph of the Abstract to further emphasise limitations (see Comment 2).

Please note that finding no evidence that PrEP reduces condom use is not, essentially, inconclusive. It is a finding inconsistent with the hypothesis of risk compensation. The caveat, which we are careful to acknowledge, is that the finding could possibly reflect limited power or potential bias.

6. Also, the reporting should be substantially improved. For example, it's unclear whether the participants gave informed consent, and the exact timing of the FSW randomization remains unclear (Zelen design?).

Response. We have edited the manuscript to describe the study design and execution with greater clarity and to improve the reporting of results. We have explicitly stated in the

manuscript that we report following CONSORT guidelines (included as Supporting Information). On the specific issue of participant consent and the timing of randomisation, we have clarified:

> "*The trial had a Zelen design [19]: informed consent was sought and obtained after randomisation. FSWs randomised to the treatment group were contacted and informed about the study's purpose, procedures, potential risks and benefits. If they consented to participate, they were given an appointment the following day for PrEP referral. Three to eight months later, all 2020 survey participants potentially eligible for PrEP (in 2020) who had been randomised to either the treatment group or the control group were invited to participate in an endline survey held in April-May 2022. Informed consent was obtained from all participants of this survey. The control group participants were never invited to participate in the PrEP intervention. They were offered PrEP as part of its general rollout in Senegal after the trial.*
>
> *Randomisation and masking*
>
> *"Prior to contacting the 2020 survey participants who were potentially eligible for PrEP, in October 2020, we randomised them to treatment and control groups (3:2)."*

Reviewer #1

[Summary omitted]

1. Did participants in the ongoing longitudinal survey consent to participating in a randomised trial in the future? If not, was a waiver of consent approval sought for this RCT?

Response. The participants in the ongoing longitudinal survey did not consent to participation in a future randomised trial, which was not planned when the cohort survey started in 2015. As explained in our response to Academic Editor, Comment 6, the RCT had a Zelen design, with consent for trial participation sought and obtained after randomisation to the treatment group. Consent for survey participation was obtained from all those surveyed. This design, including obtaining control group consent only for participation in the endline survey, was approved by the national ethics committee of Senegal and by the ethics committee of the PI's institution. Please note that the control group participants were not exposed to any intervention or additional procedures beyond those for participation in the longitudinal survey. All participants in both the treatment and control groups consented to participation in the endline survey.

2. Three primary outcomes are identified in the protocol - condom use in last two clients, condom use in last 5 clients, and subjective perceptions of condom use on a likert scale. In the manuscript, there are two primary outcomes, condom use with last client, and condom use with three last clients, with the Likert outcome moved to a secondary outcome. The online registration also mentions three different modes of capturing condom use with clients. Were the list experiment and newly designed instrument detailed in the online registration used in the end? When was the decision to change the definition of the primary outcomes from the protocol to the version in the manuscript? Was this before or after unblinding?

Response. The change in the number of clients specified in the condom use primary outcomes was made prior to unblinding. Condom use with clients was one of several questions about sex acts, client characteristics and the context of an encounter with a client. Prior to finalizing the questionnaire and fielding the endline survey, we realised that it would be overly burdensome to ask participants to answer all these questions for each of their last five clients. To reduce the survey burden, we decided to ask about encounters with the last three clients.

With data available on the last three clients, we decided to define primary outcomes as condom use with the last client and with all of the last three clients in order to cover the full

range of data available. The protocol specified condom use with **each** of the last two clients (as well as the last five clients, which was no longer feasible). That is, the last client and the penultimate client. We added supplementary analysis to estimate effects on condom use with the penultimate client. We edited the Methods, Outcomes sub-section to mention this outcome, summarised the results using it in Results and provided the respective estimates in **SM Table S8**. The estimates are very similar to those obtained for condom use with the last client and do not change the main study findings. In the manuscript, we added:

> "*The estimated 95% CIs for effects of PrEP referral and PrEP use on condom use with the penultimate client all include zero (**SM Table S8**).*"

We also elicited condom use with the two indirect methods referred to in the protocol (double list experiment and colourbox methods). We had three reasons for deciding not to estimate and report effects on condom use elicited with these methods.

First, condom use prevalence estimated with the new *colourbox* elicitation method was very similar to the estimate obtained with direct elicitation. This suggests that either there was little misreporting of condom use or the method was not effective in correcting any misreporting. Either way, we realized that the effect of PrEP on colourbox-elicited condom use was unlikely to differ from the estimated effect on direct-elicited condom use. Prior to submission, we did not estimate effects using the colourbox-elicited outcome.

Second, low power caused us to drop the double list experiment-elicited outcome which was only elicited for condom use with the last client due to survey burden. This method embeds an elicitation experiment within the PrEP intervention experiment. Hence, analysis using the list experiment outcome would have even less power than that using the directly elicited outcome. Prior to submission, we did not estimate effects using the list experiment-elicited outcome.

Third, given that we expected that estimates obtained with the two indirectly elicited outcomes would either differ little from those obtained with directly elicited outcomes or be inefficient, we judged that taking space to explain these (unfamiliar) methods would only divert the attention of readers with no substantive gain in terms of the evidence delivered. To maintain clarity, we decided not to run and report analysis with the indirectly elicited outcomes.

While we remain convinced of this logic, we appreciate that deviation from the protocol is, in general, ill-advised and may arouse suspicion. Therefore, we have now done the analysis for a) directly elicited condom use with the last two clients, b) colorbox-elicited condom use with the last client and the last three clients and c) list-experiment elicited condom use with the last client (this method was not used with each of the last three clients to reduce survey burden). The results, reported in **SM Table S8**, are summarised in the manuscript as follows:

" Control group prevalence of condom use with the last client was estimated to be 15.5 pp lower using the double list experiment rather than direct elicitation ***(SM Table S8)****. The point estimate of the effect of PrEP referral on the double list experiment estimate of this prevalence is positive and the 95% CI includes zero (0.011, 95% CI: -0.025, 0.060; p=0.548)* ***(SM Table S8)****. The mean ITT estimates of the two lists differed substantially, even though each had very wide confidence intervals (List 1: -0.145 [-0.530, 0.240]; List 2: 0.145 [-0.248, 0.530]). The colorbox estimates of condom use prevalence are close to those obtained with direct elicitation* ***(SM Table S8)****. Point estimates of PrEP effects on colorbox-elicited condom use are positive with 95% CIs that always include zero* ***(SM Table S8)****."*

While not stated in the protocol, condom use was always intended to be the main outcome, with subjective perceptions of HIV/STI risks (with/without condom use) a potential mechanism through which any effect on this outcome may materialise. With no negative effect of PrEP on condom use, the estimated effects on subjective risk perceptions are, arguably, less interesting. For this reason, we included these estimates in the same table as the secondary outcomes in the initial submission. We appreciate that this created an inconsistency with the protocol. We have moved the estimated effects on risk perceptions to Table 2, alongside the effects on condom use.

3. Missing data in this study is treated with a complete-case analysis, i.e. assuming that data is 'missing completely at random', and with a relatively large number of participants who missed the follow-up survey. An additional analysis that considers the data to be 'missing at random' (e.g. IPW, MI) should be considered in a revision.

Response. We added a supplementary analysis using entropy balancing to reweight the complete-case, post-attrition analysis sample to match covariate moments of the pre-attrition sample. The main finding of no negative effect on condom use is robust to this sample reweighting. We have added the results of this analysis as SM Table S6 and summarised them in the Results sub-section:

*"Estimates of the effects on condom use are similar … with reweighting to match, on moments of covariates, a) the analysis sample to the pre-attrition sample … (****SM Table S6****).*

In the Statistical Analysis sub-section, we added:

"In supplementary analysis, we estimated effects on the condom use outcomes …with entropy balancing [27] used to reweight and match, on moments of covariates, a) the (post-attrition) analysis sample to the pre-attrition sample …."

4. One of the limitations of the study is that study participants were not specifically recruited for the RCT and are randomised before they are contacted for the study (similar to a Zelen's design or Trial Within Cohort). I would also recommend a sensitivity analysis of a 'missing-not-at-random' scenario for any post-randomisation differential effect of the PrEP contact/visit on follow-up (e.g. controlled imputations with delta adjustment or a tipping point analysis).

Response. We have clarified in the Methods and Findings section:

> *"The trial had a Zelen design [19]: informed consent was sought and obtained after randomisation."*

In the Results sub-section, we reported that selection into the analysis sample did not differ by treatment assignment (p-value = 0.6, **SM Table S3**). Further, we reported that, in the analysis sample, the treatment and control groups were balanced on all but two baseline characteristics and the null of no differences across multiple characteristics was not rejected (**Table 1**). Nevertheless, we adjusted for covariates. We have added results from a supplementary analysis in which we estimated effects on condom use outcomes without covariate adjustment (**Table S6**). Similarity with the adjusted estimates provides further reason to believe that there is not an important bias due to any differential attrition between the treatment and control groups. To provide further reassurance, we added another supplementary analysis (**Table S7**) that used entropy balancing to match the treatment group to moments of covariates of the control group in the analysis sample. The estimates are also robust to this reweighting.

In the Statistical Analysis sub-section, we added:

> *"In supplementary analysis, we estimated effects on the condom use outcomes without covariate adjustment and with entropy balancing [27] used to reweight and match, on moments of covariates, … b) the treatment group to the control group in the analysis sample. "*

In the Results sub-section, we added:

> *"Estimates of the effects on condom use are similar without adjustment for covariates (**SM Table S6**) and with reweighting to match, on moments of covariates, … b) the treatment group to the control group in the analysis sample (**SM Table S7**)."*

In the Limitations sub-section, we added:

> *"Fourth, participants were not specifically recruited for the RCT and were randomised before attempts were made to contact those assigned to the treatment group for consent, leaving scope for differential study participation between the treatment and control groups, which is a particular concern given a high rate of attrition (49%). However, we showed that treatment assignment had no effect on study participation,*

we adjusted for covariates and demonstrated robustness of the estimated effects on condom use to this adjustment and to matching the analysis sample to the pre-attrition sample and the treatment group to the control in the analysis sample."

5. Page 7, Paragraph 1. Were the two primary outcomes (last client and last three clients) considered co-primary or multiple outcomes? E.g. was 'success' of the trial considered to be effect in both outcomes, or either outcome?

Response. We revised the text to read as follows:

> *"The main objective was to determine whether PrEP affected condom use with clients. The primary outcome was condom use with clients self-reported in the endline survey. We asked each participant to recall their last three clients and whether a condom had been used with each of them (**SM Table S1**). We created two primary binary outcomes: a) 1 if a condom was used with the last client, and 0 otherwise; b) 1 if a condom was used with all three clients, and 0 otherwise. These were multiple outcomes: a negative effect on either would be of concern."*

6. Were study staff collecting the follow-up data blinded to the treatment allocation?

Response. They were partially blinded. Endline survey enumerators were not explicitly told the treatment assignment of each participant. Importantly, when asking about primary outcomes — condom use and risk perceptions – they did not know the assignment. After these outcomes were recorded, there was a question to PrEP users only: "Who invited you for PrEP?". The response revealed any PrEP user who had been assigned to the treatment group. The treatment of assignment of non-users of PrEP could not be determined. This procedure was followed to minimize potential bias in the measurement of primary outcomes while meeting the need for information on PrEP users experience specific to the intervention.

We clarified in the Methods and Findings, Outcomes sub-section:

> *"Enumerators were blind to treatment assignment when recording the primary outcomes — condom use and risk perceptions. After measurement of these outcomes, a question only posed to PrEP users revealed the treatment assignment of those participants, but not that of PrEP non-users. The midwives and peer facilitators who recruited FSWs to the endline survey, but who were not enumerators, also recruited to the PrEP intervention, and so were not blinded to treatment assignment. "*

7. Page 8, Paragraph 3. What covariates were used in the adjusted regression

models? Are these the ones listed on page 9? Are the same variables used in every analysis reported? What was the rationale for including these covariates?

Response. We clarified in the Statistical analysis sub-section:

> *"Covariate adjustment was done to control for any treatment-control group imbalance that may have resulted from attrition, to increase precision, and, in estimation of effects of PrEP use, to control for systematic non-compliance. The covariates used in all analyses were age (years) in 2022, number of days in the seven preceding the endline interview that were within Ramadan, and 2020 values of marital status, FSW registration status, self-reported sexual risk taking, prior PrEP experience and the respective (lagged) outcome."*

As mentioned above, the main findings are robust to not adjusting for covariates.

8. Could a further explanation be provided about the estimated effect - does the 'outcome probability averaged over the sample' mean that the observed margins were used instead of reference values?

Response. We have clarified:

> *"For each binary outcome, we report the estimated risk difference (RD), which is the estimated effect of PrEP referral on the marginal outcome probability for each participant (at observed covariates) averaged over the sample."*

9. Page 10, Paragraph 3. I would remove the test of baseline characteristics, as the only way to see a 'significant' difference is by chance (in Table 1 as well). CONSORT makes specific recommendations to not report these.

Response. Table 1 reports on baseline characteristics of the complete-case, post-attrition analysis sample. Differences between treatment and control groups could emerge through differential attrition. Hence, it seems appropriate to test between-group equality of means of these characteristics. We removed the tests in Table S1 for the pre-attrition sample and have dropped reference to the respective p-value (of the joint test) in the text, which now reads:

> *"IIn the (post-attrition) analysis sample, the treatment and control groups were balanced on baseline characteristics except for differences in marital status and client numbers. A joint F-test did not reject the null hypotheses of no differences across the multiple characteristics (p-value=0.275). There was also balance in the pre-attrition sample (SM Table S2)."*

Reviewer #2

[Summary omitted]

Major comments

1. There was quite substantial attrition in the trial (both survey and sex work attrition), which I would like to see more clearly highlighted in the article. In the results, where you report the numbers excluded for these reasons, please also present the % excluded for these reasons. In the discussion, instead of mentioning the high attrition rate and consequent reduced power at the end of a point about future studies, I would like to see the high attrition listed explicitly as a limitation at the start of one of these points.

Response. We have edited the Results sub-section to provide the percentages:

> "Out of 604 FSWs in the sampling frame (surveyed in 2020), 500 (82.8%) were assessed, based on 2020 data, as potentially eligible for PrEP and were randomised to active PrEP referral (treatment, n=300) or delayed (until after endline) PrEP referral (control, n=200) (Figure 1). Out of the 500 FSWs randomised, 308 (61.6%) were included in the analysis sample, with 95 (19.0%) excluded because they could not be traced or they refused to participate in the endline survey and 97 (19.4%) excluded because they reported not doing sex work at the time of the survey and, hence, sex work outcomes could not be elicited."

In the Limitations sub-section, we added:

> *"Fourth, participants were not specifically recruited for the RCT and were randomised before attempts were made to contact those assigned to the treatment group for consent, leaving scope for differential study participation between the treatment and control groups, which is a particular concern given a high rate of attrition (49%). However, we showed that treatment assignment had no effect on study participation, we adjusted for covariates and demonstrated robustness of the estimated effects on condom use to this adjustment and to matching the analysis sample to the pre-attrition sample ... "*

Please see responses to related Reviewer 1, Comments 3 and 4.

2. P20, paragraph 4: 'Table 4 shows estimates of effects of PrEP referral and PrEP use on beliefs about the effectiveness of condoms in preventing HIV and STIs.' Should this be Table 3? Please check. Similarly, later in that paragraph where you refer to SM Table S5, I think this should be SM Table S4. At the end of the paragraph, where you mention 'Estimates of effects of PrEP referral on beliefs about the effectiveness of condoms in preventing other STIs do not show the same consistency, have much

larger p-values and much smaller effects', please add references to the relevant tables showing this information (Table 3 and Table S4).

Response. Apologies. We have corrected the mistakes and made explicit reference to the table and supplementary table.

3. P21, paragraph 2: '9% of PrEP users answered that they are likely or very likely to being infected by HIV when using PrEP only versus 20% for condom only' I think these are labelled the wrong way round, so it should be '9%... when using condoms only versus 20% for PrEP only'. Please check and correct.

Response. We have corrected the error and rewritten the whole paragraph summarising Table 4 to improve clarity.

> *"Table 4 shows that only 6% of PrEP users reported believing that they would be very unlikely to be infected with HIV if they were to have sex with someone who was HIV-positive and they were neither taking PrEP nor wearing a condom. Theis percentage describing the risk of HIV infection as very unlikely increased to 38% and 40% if they were to use either PrEP or a condom, respectively. And 91% of PrEP users stated that they would be very unlikely to become infected if they were to use both PrEP and a condom. Among PrEP users, 20% assessed the risk of HIV infection as likely or very likely if they were to only use PrEP, compared with only 9% who rated the risk at this level if they were only to use a condom."*

Minor comments

1. There are minor spelling and grammatical errors throughout. A few are listed below, but I suggest a thorough proof-reading of the article before resubmission

a. Abstract: Background: generates negative -> generate negative

b. P14, Methods: 'and full protocol is available on the UCL repository' -> and the full protocol is available in the UCL repository

c. P14, Study design and participants: 'excluding those who (a)…' there is no corresponding '(b)'

d. P15, Intervention: 'Those who eligible' -> 'Those who were eligible'

Response. We have corrected these errors and others found in a thorough proof-reading of the paper.

2. Abstract, Findings: for consistency, please include the p-value for final set of results presented (effect of PrEP use on condom use with all 3 last clients), and please include 'pp' as units for this

Response. Done.

3. You mention several times that midwives recruited participants - was it really midwives rather than nurses? Midwives assist women in childbirth, so it seems unlikely that they would have a recruitment role in a PrEP trial.

Response. It is accurate that midwives, rather than nurses, were involved, as they are responsible for the follow-up component of Senegal's compulsory registration law for FSWs. Under this law, FSWs are required to undergo monthly pelvic examinations conducted by midwives. Leveraging their established relationships and trust with the participants, midwives were engaged to assist with PrEP recruitment. We added:

> "*By law, midwives are responsible for follow-up with registered FSWs.*"

4. P16, Outcomes: for the scale eliciting beliefs about the likelihood of contracting HIV/STI, it is unclear how the information in a) (with and without use of a condom) differs from the information obtained from b) and c) together. It may be helpful to reproduce the questions in the appendix?

Response. We have added **SM Table S1** to provide the questions. In the Outcomes sub-section, we have clarified:

> *"We used a 5-point Likert scale (1: Very unlikely, ... 5: Very likely) to elicit perceptions of the risk of contracting HIV (STI) through sex with someone with HIV (STI) a) if a condom were not used and b) if a condom were used (SM Table S1). For each of HIV and STI, we created a binary outcome equal to 1 if the perceived risk was at or above the median response (= 5) in a): High risk without condom. Another binary outcome was 1 if the perceived risk was not above the median response (= 1) in b): Low risk with condom. We created a third outcome equal to 1 if both of the other two were equal to 1: High risk without & low risk with condom.*

Moved description of the other measure of HIV risk to Statistical Analysis because it is not used as an outcome. THe relevant text is:

> "*We asked only PrEP users to report on a 5-point Likert scale (1: Very unlikely, 5: Very likely) their perceptions of the risk of contracting HIV through sex with someone with HIV if they were to use a) no PrEP and no condom, b) no PrEP but a condom, c) PrEP but no condom, and d) both PrEP and a condom."*

5. P16, Pre-experiment power calculation: the reader may not know what list experiments are. These are explained further in the discussion, but it may be helpful to clarify here, or add some information in an appendix which can be referred to here

Response. We have now explained the list experiment in the Outcomes sub-section and referred to the new **SM Text S2** that provides a fuller description of the method before mentioning it in the power calculation.

6. P19/p20: is 'recently used PrEP' the same as 'having used PrEP in 2021/22'? If so it would help to define recently used PrEP as this, or even to use the same phrase throughout.

*Response***.** We now define the two PrEP utilisation measures explicitly in Procedures section, give them labels and then use these labels consistently.

> *"Participants were asked: "Are you currently on PrEP to prevent HIV?". We coded those responding positively as 1 for the binary indicator* ***PrEP use****. Participants were also asked: "Have you started taking PrEP this year or last year?". We coded those responding positively as 1 for the indicator* ***Ever used PrEP in past year****."*

7. P20, paragraph 2: 'Table 2 shows estimates of ITT effects of PrEP referral, effects of PrEP use on condom use and effects of having used PrEP in 2021/2022.' Are the effects all on condom use? It's a bit unclear from this sentence.

*Response***.** We edited the sentence to read:

> *"Table 2 shows estimates of effects of PrEP referral and the two measures of PrEP use on condom use and on perceptions of HIV and STI risks."*

Please note that in response to this Reviewer 1, Comment 2, we now include the estimated effects on HIV/STI risk perceptions in Table 2.

8. P20, paragraph 3: 'alternative outcome specification' - it would helpful to briefly summarise what this was.

Response. We clarified:

> *"However, this finding is not robust to estimating the effect on a continuous linear measure derived from this categorical variable (****SM Table S9****) rather than estimating the effect on a binary measure."*

Prior to this, in Statistical Analysis, we clarified:

“In supplementary analysis, we treated these categorical variables as continuous linear measures (e.g., scaled 1-5) …”

9. P21, Discussion, paragraph 1: '…among PrEP users and FSWs recently exposed…' for clarity, I suggest changing this to '…among PrEP users or among FSWs recently exposed'

Response. We have completely rewritten this paragraph.

10. P21, Discussion, paragraph 1: 'When taking all three clients together' I suggest clarifying to say '…all three most recent clients together'

Response. We have done this.

11. P23, last paragraph: 'The latter method…' do you mean the colorbox method? Please make clear.

Response. We have rewritten this part.

12. References: reference 1 gives an out-of-date link. For preference, use a peer-reviewed article as a source for this information.

*Response***.** We replaced this reference with references to peer-reviewed articles.

Jourdain H, de Gage SB, Desplas D, Dray-Spira R. Real-world effectiveness of pre-exposure prophylaxis in men at high risk of HIV infection in France: a nested case-control study. The Lancet Public Health. 2022 Jun 1;7(6):e529-36.

Murchu EO, Marshall L, Teljeur C, Harrington P, Hayes C, Moran P, Ryan M. Oral pre-exposure prophylaxis (PrEP) to prevent HIV: a systematic review and meta-analysis of clinical effectiveness, safety, adherence and risk compensation in all populations. BMJ open. 2022 May 1;12(5):e048478.

Reviewer #3

Introduction

1. The PrEP landscape is rapidly evolving with multiple PrEP options. Please consistently specify the modality (oral) of the PrEP referenced.

Response. In the Abstract, Author Summary, Background and Methods and Findings, we have clarified that the intervention is referral for oral PrEP.

2 The authors mention that participants gave consent to each survey, but it is not clear if the participants also provided consent to the RCT?

Response. Please see responses to Editor, Comment 6 and Reviewer 1, Comment 1.

3. Randomization was stratified by PrEP experience and sexual risk-taking behavior, which makes sense. Can the author briefly state their rationale in the text?

Response. Thank you for raising this point. We have now revised the manuscript to include this explanation:

> "*Randomisation was stratified by prior experience with PrEP and sexual risk-taking both self-reported in the 2020 survey (Supplementary Material (SM) Text S1). Prior PrEP experience would have been gained through a discontinued PrEP demonstration project in 2015/16 [24]. Any behavioural response to the present study's intervention could vary with prior PrEP exposure and propensity for risky sex behaviour, which was the reason for stratifying on these characteristics*."

4. It is not clear when the randomization occurred? Please clarify.

Response. In response to comments above, we revised the text to make clear that the RCT had a Zelen design, with randomisation prior to seeking consent. On the specific timing of randomisation, we clarified:

> *"Prior to contacting the 2020 survey participants who were potentially eligible for PrEP, in October 2020, we randomised them to treatment and control groups (3:2)."*

5. Additional details of randomization process are needed. Who was blinded to groups assignment? Were the staff who administered the endline survey blinded? Where the staff who contacted the participant in the intervention arm blinded? Or what measures were put in place to minimize bias in assessment?

Response. Please see response to Reviewer 1, Comment 6. We clarified in the Methods and Findings, Outcomes sub-section:

> *"Enumerators were blind to treatment assignment when recording the primary outcomes — condom use and risk perceptions. After measurement of these outcomes, a question only posed to PrEP users revealed the treatment assignment of those participants, but not that of PrEP non-users. The midwives and peer facilitators who recruited FSWs to the endline survey, but who were not enumerators, also recruited to the PrEP intervention, and so were not blinded to treatment assignment. "*

6. The intervention only lasted for 4 months, from October 2021 to January 2022, while there are two years between the two waves of the survey. HIV risk and exposure is a dynamic and not static process that there can be a lot of changes even in a space of 3 months. Can the author clarify their rationale for conducting only a 4-month PrEP referral program during the two-year follow up period?

Response. Apologies for the impression that the study had a two-year follow-up period. We have clarified the timing as follows.

> Study design and participants
>
> *"The sampling frame was a list of participants of a survey of FSWs conducted between June and August 2020 in Dakar, Senegal…..*
>
> *The 2020 survey was not a baseline for this study. It provided the sampling frame and some data used for stratification and controls."*
>
> Randomization and masking
>
> *"Prior to contacting the 2020 survey participants who were potentially eligible for PrEP, in October 2020, we randomised them to treatment and control groups (3:2)."*
>
> Procedures
>
> *"The intervention was referral for oral PrEP in Senegal. It was planned that rollout would begin soon after randomisation in October 2020, but it was delayed for various reasons. From September 2021 to January 2022, midwives at public health centres and FSW peer facilitators actively contacted FSWs assigned to the treatment group and asked if they were interested in receiving PrEP."*
>
> *"The primary source of data is the endline survey, which was fielded in April-May 2022, three to eight months after the treatment group was referred for PrEP."*

There was an unforeseen delay in implementation of the intervention and between randomisation and implementation. But the study design does not have a 2-year follow-up.

Evaluation is based on outcomes measured at endline, three to eight months after exposure to the intervention.

7. Additional information about the intervention is needed? How were the midwives and FSW facilitators trained in referring FSWs to PrEP? How often did they reach out, and in what way? If a participant showed no interest in PrEP, would they receive another contact? How long did it typically take from referral to PrEP screening?

Response. We have clarified in Methods, Intervention:

> *"Midwives and FSW facilitators received comprehensive training from the HIV Division of the Ministry of Health that developed the national PrEP protocol. The training included guidance on counselling, referral processes, and addressing potential barriers to PrEP uptake. Eligible participants were contacted by phone by a midwife or a FSW facilitator, who provided information about PrEP and its benefits. Participants who expressed interest and provided verbal consent were invited for PrEP eligibility screening on the following day at one of the designated referral sites*.'

8. For data collection, can the author add more details about the variables collected? For example, how was PrEP utilization assessed in the survey?

Response. We have rewritten the sections on Methods and Findings, Procedures, Outcomes and Statistical analysis to clarify measurements of exposures, outcomes and controls. Please see responses to Reviewer 1, Comment 7 and Reviewer 2, Minor Comments 4 and 8. Specifically on the measurement of PrEP utilisation, we have added in Procedures:

> *"Participants were asked: "Are you currently on PrEP to prevent HIV?". We coded those responding positively as 1 for the binary indicator* ***PrEP use****. Participants were also asked: "Have you started taking PrEP this year or last year?". We coded those responding positively as 1 for the indicator* ***Ever used PrEP in past year****. These self-reports of PrEP utilisation were cross-checked, as far as possible, with MoH and ANCS records."*

9. **The authors examined PrEP use in two ways: current use at the time of the endline survey and recent PrEP use but there is no clear description of this classification is provided. Can the authors define recent PrEP use? Based on the results section, recent PrEP use refers to PrEP use in 2021 and 2022. Can the author justify why this period was used? Why was PrEP use during the entire follow-up period or after the start of the PrEP referral program not measured?**

Response. Please see the response to the previous comment. We use two measures of PrEP utilisation because a participant may stop using PrEP, and past use may still affect current behaviour (e.g. condom use). As explained in the third paragraph of the Procedures section, we can only use the endline survey to consistently (for treatment and control groups) measure PrEP use. The survey asked about current use and use in the past year. We use all these data. Please again note that we do not have a follow-up period from the 2020 survey until the endline survey. PrEP use in the past year from the endline survey is sufficient to capture use induced by exposure to PrEP referral that occurred 4-8 months earlier. .

10. Please clarify what "exposure to Ramadan within 7 days" refers to?

Response. We clarified in the Statistical analysis sub-section that we control for:

> "... *number of days in the seven preceding the endline interview that were within Ramadan, …"*

10. Can the authors specify more details on the inclusion and exclusion criteria for the actual analysis?

Response. Above the Participant Flow in Figure 1, we have added:

> *"Out of 604 FSWs in the sampling frame (surveyed in 2020), 500 (82.8%) were assessed, based on 2020 data, as potentially eligible for PrEP and were randomised to active PrEP referral (treatment, n=300) or delayed (until after endline) PrEP referral (control, n=200) (Figure 1). Out of the 500 FSWs randomised, 308 (61.6%) were included in the analysis sample, with 95 (19.0%) excluded because they could not be traced or they refused to participate in the endline survey and 97 (19.4%) excluded because they reported not doing sex work at the time of the survey and, hence, sex work outcomes could not be elicited."*

11. The intervention was referral for PrEP. So, there was no attempt for any form baseline assessment for the control group. A design in which the evaluation between the two groups is not similar always bound to introduce bias. Please justify.

Response. In response to this comment and related comments from the Editor and other reviewers, we clarified that the RCT had a Zelen design – with consent sought from the treatment group only after randomisation – acknowledged that this design is potentially vulnerable to bias through differential attrition between the treatment and control groups, and we added additional supplementary analyses to assess and reduce the risk of bias. Please

see responses to Editor, Comments 2 & 6, Reviewer 1, Comments 1 & 4 and Reviewer 2, Comment 1.

12. The RCT was designed to evaluate the effect of the 4-month PrEP referral program on changes in sexual behaviors among FSWs. However, the question the presented in analysis presented by authors aimed to answer focuses on PrEP use and sexual behaviors, which is not the primary question of the study.

Response. The RCT was not designed only to evaluate the effect of PrEP referral. The central question that motivated the study is whether use of PrEP, which reduces the risk from unprotected sex, causes risk compensatory behaviour, e.g. more unprotected sex. The RCT was designed to induce exogenous variation in PrEP use through random assignment to PrEP referral. In other words, the RCT was an encouragement design experiment (e.g. see https://link.springer.com/article/10.1023/A:1008045618501). We have now stated the study objective more clearly:

> *"This study aimed to use the randomised rollout of oral PrEP to FSWs in Senegal to obtain the first causal evidence on whether there is risk compensation in response to use of this effective HIV preventive medication in a high-risk population."*

We updated the Methods and Findings, Statistical Analysis sub-section to clarify how this objective is realised:

> *"We estimated effects of PrEP use and Ever used PrEP in past year on the outcomes. To estimate these effects allowing for non-random variation in use resulting from two-sided noncompliance, we used exogenous variation in each measure of PrEP use that was generated by random assignment to PrEP referral. That is, we used an encouragement design [26] in which assignment to the treatment or control group was effectively used as an instrument for PrEP use, ensuring identification of the effect from response of the respective outcome only to experiment-induced random variation in use."*

13. In an intent-to-treat analysis, participants are analyzed based on the group they were randomized to regardless of whether they actually participate or not in the assigned group or not. So, the primary which focus on 182 vs 126, is modified ITT

Response. We revised to:

> *"We estimated modified intention-to-treat (ITT) effects of PrEP referral on the outcomes. Modification was due to randomisation prior to seeking consent and loss to follow-up because of attrition from sex work and the survey."*

14. For the FSW population, as the probability of condomless sex is already high. Did authors consider focus on analysis focused change in trends or change from baseline analysis?

Response. In the study protocol, we specified that the 2020 survey would be used as a sampling frame, and not as a baseline from which to measure outcome changes. One reason was the intention to use the colourbox method to elicit the primary outcome – condom use. This method was not used in the 2020 survey. However, in the endline survey, the colourbox method gave a very similar estimate of condom use prevalence as direct elicitation. For reasons explained in response to Reviewer 1, Comment 2, we decided to report estimates obtained with directly-elicited condom use, although we have added estimates with indirect elicitation, including the colourbox method, in this revision (**SM Table S8**).

Even without consideration of the analysis specified in the protocol, we believe it is better to estimate effects using the endline measurement of each outcome, with control for its 2020 value, rather than use the change from the 2020 value. As explained in response to this reviewer's Comment 6, delay in PrEP rollout resulted in a longer than anticipated gap between the surveys. The 2020 survey did not provide a measurement of the outcome immediately before exposure to treatment. It is a noisy measure of the pre-treatment outcome. Estimation with the difference from that noisy measure would be less efficient (McKenzie 2012). Given that power is already an issue, the ANCOVA analysis (controlling for the 2020 outcome value) we used is likely to give a more reliable estimate.

Furthermore, the outcome measures in the two waves do not always match up. The 2020 survey was carried out to study a wide range of topics. It was not designed as a baseline for the RCT we report here. For example, in the 2020 survey, condom use was elicited for the last two clients, while in the endline survey, which was conducted specifically for this RCT, condom use was elicited for the last three clients.

Finally, note that while prevalence of reported condom use was high in the 2020 survey, it was much lower for the control group in the endline survey.

McKenzie, D. (2012). Beyond baseline and follow-up: The case for more T in experiments. *Journal of Development Economics*, 99(2), 210–221.

15. Similar to my comment above, this is a longitudinal study but there was no attempt assess the change in risk behaviors, given that the main question is about risk compensation (i.e., change in risk)?

Response. Please see our response to the previous comment. Further, the aim was to test for an effect of PrEP, which reduces the risk from unprotected sex, on that behaviour. Even

with only post-treatment measurement of the behaviour (condom use), an RCT design is sufficient to identify the effect of treatment on behaviour. As explained in response to this reviewer's Comment 12, we used an encouragement design to identify the effect of PrEP use on condom use.

16. It seems the analytic implicitly assumes that the covariance of the measured matrices to be homogeneous in the two groups NOT heterogeneous which seems be an oversimplification given the differences in outcome assessment for treatment vs control. Indeed, if there is heterogeneity in variance across groups, repeated measures analysis utilizing Kenward and Roger's adjustment would be preferred. Please provide justification for your approach.

Response. For inference, we used (heteroscedasticity-consistent, HC) robust standard errors (White 1980) that allowed for unequal variances and accounted for heterogeneity without assuming the form of such heterogeneity. We have clarified this when we first mention the use of robust standard errors.

> *"In this analysis, and all others, used robust standard errors, which allowed for any form of unequal variances between groups, for inference.[White 1980]"*

Please see our response to the reviewer's Comment 14 for the reason that we did not use repeated measures analysis.

White, Halbert (1980). "A Heteroskedasticity-Consistent Covariance Matrix Estimator and a Direct Test for Heteroskedasticity". Econometrica. 48 (4): 817–838. doi:10.2307/1912934

17. In the power estimation, an attention rate of 10% was assumed, However, from the n=500 randomized individual to final analytic sample n=308, it represents nearly 40% attrition and for the true ITT sample n=415, it's ~17% attrition. It possible that the observed null effect could be due to type II error? Can the authors do power simulation based in the final sample size to estimate the available power? Also, what were the characteristics of those no included in those two samples compared to those not included in the analysis?

Response. While post-hoc power analysis is generally discouraged, we appreciate the reviewer's concern that low power could reduce the information content of the null effect for condom use with the last client. To assess the available power, while avoiding the pitfalls of post-hoc analysis, we used the MDE calculated before data collection to back out the post-hoc power. In doing this, unfortunately, we found a mistake in the reporting of the previously calculated MDE. We have corrected this:

"With 80% power and a two-sided two-sample proportion test (alpha=0.05), for condom use with the last client, we calculated the intention to treat MDE of PrEP referral as 13.1 percentage points (pp) and the MDE of PrEP use as 16.8 pp."

Using the corrected MDE, the post-hoc power for the effect of PrEP use on condom use with the last client is only 24%. For the effect on condom use with all of the last three clients the post-hoc power is stronger, at 62%. The calculations are provided below.

Since low power can also result in a false positive result, we calculated the probability that the statistically significant estimate of the ITT effect on condom use with all of the last three last clients has the wrong sign (Type S error) (Gelman and Carlin, 2014). That is, we calculated the probability that we failed to detect a true negative effect of PrEP on condom use consistent with risk compensation. The probability of such an error is low, at only 0.3% (calculations below).

We have acknowledged the low power, and advised that evidence from this study be supplemented with evidence from larger samples or used in a meta-analysis, in the Abstract, Author Summary and the Limitations sub-section. In the latter, we added:

"This study had several limitations. First, it was underpowered for precise estimation of effects on the condom use outcomes. A post-hoc power analysis based on the ex ante MDE revealed that, for PrEP use, there is only 24% power for estimation of the effect on condom use with the last client, but 62% power for the effect on condom use with all of the last three clients. However, we calculated that there is only a 0.3% chance that the statistically significant estimate of a positive ITT effect of PrEP on condom use with all of the last three clients has the wrong sign (Type S error)[28], which means it is highly unlikely that the study failed to detect true risk compensation behaviour of reduced condom use in response to PrEP. Nonetheless, the finding that PrEP did not reduce condom use should be confirmed by studies with larger samples or the estimate from this study should be combined with other estimates in a meta-analysis.

Lower-than-expected power was partly due to delayed PrEP rollout that did not start, as scheduled, soon after the 2020 survey. This increased survey and sex work attrition. Two-sided non-compliance was also higher than anticipated. Actual PrEP take-up in the treatment group rate was much lower than the 82.4% seen in a PrEP demonstration study [19]. Furthermore, while study partners were asked to delay PrEP rollout in the control group until after the endline, this proved hard to implement. Nonetheless, randomised PrEP referral did substantially increase PrEP use, which made it possible to estimate the behavioural response, and we used methods that ensured that this effect was identified only from the experiment-induced random variation in PrEP use. Further, null effects on secondary outcomes are consistent with no marked behavioural response to PrEP, which gives greater confidence that

the finding of no negative effect on condom use was not merely attributable to low power."

In response to the question of how the characteristics of those not included compare with those of those included in the analysis sample, please see SM Table S3 that shows that sample inclusion was associated with some covariates but, importantly, not with treatment assignment. Also note that, in response to Reviewer 1, Comment 3, we added supplementary analysis in which we reweighted the analysis sample to match the pre-attrition sample on moments of covariates (SM Table S7). The results are robust. They are also robust to matching the treatment group to the control group in the analysis sample (SM Table S7). All of these findings suggest that there is little or no bias from selective attrition.

Calculate post-hoc power using original MDE

We originally powered the study using condom use with the last client. Assuming the original PrEP use MDE of 16.8 pp, the post-hoc power of condom use with the last client is 24.5% (without covariate adjustment). This reduction in power is predominantly driven by low two-sided non-compliance, i.e. 34.5 percent of the sample has to drive a similar ITT estimate instead of the 78.3 percent originally assumed. We initially did not compute power for the the effect on condom use with the last three clients as we only had data the last two clients from the 2020 survey. Using the original PrEP use MDE of 16.8, we approximate the post-hoc power of condom use with all of the last three clients as 62.4% (without covariate adjustment).

(A) Outcome 1: Condom use with last client

Step 1: Use original MDE for PrEP use 16.8%

Step 2: Calculate what PrEP referral ITT estimate has to be given a compliance rate of 34.5% → PrEP referral ITT estimate should reduce by 16.8% * 34.5% = 5.8% as only 34.5% of the sample is assumed to see a 16.8% change in behaviour

Step 3: Use actual endline survey's control group mean of 85%, final control group sample size of 126 and treated group sample size of 182.

Step 4: Key inputs into STATA command to calculate power for a two-sided test:

power twoproportions 0.85, n1(126) n2(182) diff(-0.05782321)

Power = 24.5%

(B) Outcome 2: Condom use with all last three clients

Step 1: We calculate an adjustment factor. This adjustment factor is used to account for the fact that condom use with *N* clients is not just condom use with one client * *N* as these events are not independent. Given condom use with last client is 85% and condom use with all last three clients is 67.5%, we calculate an adjustment factor (adj) as such:

P(condom use with last client) * P(condom use with penultimate client | condom use with last client==1) * P(condom use with third last client | condom use with last client==1) = 0.675

→ P(condom use with last client) * [adj * P(condom use with last client)]^2 = 0.675

→ adj = 1.05

Step 2: From (A) Step 2, original MDE for PrEP use 16.8% implies a drop of 5.8% for PrEP referral ITT for condom use with last client. We apply this drop to the equation above to translate condom use with last client to condom use with all three last clients.

(P(condom use with last client) - 0.058) * [adj * (P(condom use with last client) - 0.058)]^2 = 0.546

Step 3: We input the expected mean difference for PrEP referral ITT for condom use with all last three clients (54.6% - 67.5% = -12.9%) into the STATA command to calculate post-hoc power

power twoproportions 0.675, n1(126) n2(182) diff(-0.129)

Power = 62.4%

<u>Calculation of S error</u>

We use the retrodesign package in R by Gelman and Carlin (2014).

Using the implied post-hoc ITT MDE of condom use with all last three clients (12.9%), and the observed confidence interval width (and hence standard errors) in the data, we ran the following command to get the S error (sign error):

retro_design_closed_form(-0.129 , s=0.412/(1.96*2)) # S error: 0.3%

Gelman, A. and Carlin, J. (2014). Beyond Power Calculations: Assessing Type S (Sign) and Type M (Magnitude) Errors. *Perspectives on Psychological Science*, 9(6): 641 –651 DOI: 10.1177/1745691614551642

18. In the power estimation, attrition of 10% was assumed for what definition of attribution precisely? 10% of those selected for randomization?

Response. We clarified:

> *"We calculated the minimum detectable effect (MDE) on condom use with the last client given the fixed sample size available from the 2020 survey. We assumed a*

drop-out rate of 10% (survey and sex work attrition) from the original sample of 500, a 5% PrEP ineligibility rate,......"

19. Table 1 should present the characteristics of participants who were randomized n=500.

Response. As clarified in response to this reviewer's Comment 13, we estimate a modified ITT effect. Table 1 shows the characteristics of the analysis sample that was used to produce the estimates. Comparison of the balance of characteristics of the treatment and control groups in this sample is most relevant to assessing the evidence presented. To keep the main text streamlined, we only show the analysis sample. Characteristics of the pre-attrition sample that was randomised are given in **SM Table S2**.

20. It is not appropriate to call an analysis which excludes participants who indicated that they no longer engaged in sex work in the 2022 survey post-randomization an intent-to-treat analysis. Importantly, the action of no longer being engaged in sex work, is an outcome of the study—as it may indicate is the change HIV and thus no need for PrEP. Thus, an ITT analysis should focus on n=247 in the intervention arm vs n=158 in control arm. The arbitrary division of analysis into no longer in sex work vs still sex work non-responders leads to destruction of information and unjustified causal association interpretation. Can the author conduct an analysis by including these participants?

Response. It is not possible to include participants who were no longer in sex work because the outcomes, including the primary outcome – condom use of clients – are specific to sex work. The outcomes are not defined for those no longer doing sex work.

Please note that in response to Reviewer 1, Comments 3 and 4, we have conducted supplementary analyses that demonstrated robustness of estimates to reweighting the analysis sample to match the pre-attrition sample and reweighting the treatment group to match the control group in the analysis sample. These results build confidence that the estimates are not biased by selective attrition, including selective exit from sex work.

Again, we changed ITT to modified ITT.

21. In the second paragraph of the results section, the first sentence states that randomization occurred two years after the 2020 survey. Clarify when the randomization actually took place?

Response. Sorry, that was a mistake. In Methods, Randomization and masking, we have stated:

> “Prior to contacting the 2020 survey participants who were potentially eligible for PrEP, in October 2020, we randomised them to treatment and control groups (3:2).”

We removed the mistake in the Results sub-section, and now have:

> *“**Table 1** shows characteristics of the analysis sample at the time of the 2020 survey.”*

22. HIV risk as well as perceived risk is a dynamic process with change from time to time. Thus, to use data collected 2 years prior as the baseline may be an oversimplification of the constancy assumption. Although on average randomization would be expected to balance the group on measured/unmeasured factors, the sample size of 500 with 3:2 ratio is quite small to guarantee balance between groups especially within the key stratification categories.

Response. To help achieve balance, we stratified randomisation. SM Table S2 shows that there was indeed good balance in the pre-attrition sample of 500. Please note that we removed p-values to test equality between treatment and control groups in this sample because Reviewer 1 correctly pointed out that differences could only have arisen by chance, in any case.

The delay in rollout of PrEP contributed to high attrition due to a two-years gap between the 2020 survey, which was used as the sampling frame and for randomisation but not as a baseline (see responses to this reviewer’s Comments 6 and 14) and the endline in 2022. Despite this, we find no evidence of bias through selective attrition. Table 1 shows that the treatment and control group are well balanced in the analysis sample. We controlled for covariates (including the two used to define randomisation strata), showed that estimates were robust to not controlling for covariates, and that they were robust to reweighting the sample and treatment groups, as explained in response to this reviewer’s Comment 20.

23. What was the median time from randomization to the administration of the endline survey? How did that differ by arm. In the intervention arm, what was the median time large from referral to picking up PrEP?

Response. As noted in response to this reviewer’s Comment 21, randomisation was done in October 2020. As stated in Procedures, the endline was in April-May 2022, three to eight months after the treatment group was referred for PrEP. PrEP rollout, and hence PrEP referral, started in early September 2021. The first participant in the treatment group got PrEP on 9 September 2021. We do not have complete data on the time between referral for PrEP and receiving PrEP, but recruiters generally tried to make a clinic appointment for the next day after a participant was contacted. From the data we do have, the time between referral and receipt of PrEP was from one week to one month.

There was no systematic difference in the timing of the endline survey between the two groups.

24. What proportion of participants in the control arm had PrEP referral and/or PrEP exposure during the intervention period? And how did that affect estimates?

Response. We reported these statistics at the bottom of page 21 in the original manuscript. Proportion of participants in the control arm that had PrEP referral is 11.1% and that with PrEP exposure was 15.9%.

The uptake of PrEP in the control arm as well as the not taking up PrEP in the treatment arms are forms of non-compliance. The effects of non-compliance on the estimates are as follows:

- The column on PrEP referral is an Intention to Treat (ITT) estimate. With imperfect compliance, the ITT estimate will be closer to 0 than the case with perfect compliance.
- The columns on PrEP use and Ever used PrEP sidestep this non-compliance, as what it does is to use PrEP referral (the experimental intervention) to induce an increase in PrEP uptake and study the consequent effects of increased PrEP uptake on risky sexual behaviours. If the degree of non-compliance were to be too high, there would be an issue. Intuitively if there is little difference in PrEP uptake induced by PrEP referral, it would be difficult to study the knock-on effects of PrEP uptake on risky sexual behaviours. In our case however, PrEP referral did increase PrEP uptake and exposure quite substantially (by 34.5pp and 39.1pp respectively, also reported in page 21/22).

We added additional explanation of the latter in the Methods and Findings, Statistical analysis subsection:

> *"We estimated effects of **PrEP use** and **Ever used PrEP in past year** on the outcomes. To estimate these effects allowing for non-random variation in use resulting from two-sided noncompliance, we used exogenous variation in each measure of PrEP use that was generated by random assignment to PrEP referral. That is, we used an encouragement design [26] in which assignment to the treatment or control group was effectively used as an instrument for PrEP use, ensuring identification of the effect from response of the respective outcome only to experiment-induced random variation in use."*

25. The paragraph starting with "Table 4 shows estimates of effects of PrEP referral" should refer to Table 3 instead.

Response. Thank you for catching this error.

We have made the edit:

> *"Table 3 shows estimates of effects of PrEP referral and PrEP use on beliefs about the effectiveness of condoms in preventing HIV and STIs."*

26. In Tables 2 and 3, the reference group means for the regressions of PrEP referral, PrEP use, and ever PrEP use are different. Please specify the reference group means separately.

Response. As requested, we include a new table in the supplementary text with the outcome means among: (1) PrEP referral control group, (2) Current PrEP users and (3) Individuals who ever used PrEP in 2021/22. We added notes under this table to aid interpretation based on the respective statistical estimands shown in the main regression results tables.

Accordingly, we updated the reference group means for PrEP use in the abstract and main text, and the interpretation of the percentage effect in the main text, and have also removed the control group mean from the main regression results tables.

These are the changes we made:

Abstract

<u>Change 1: Added reference group means</u>

> *"Estimated effects of PrEP referral and PrEP use on condom use with the last client were 3.3 pp [95% CI: -4.0, 10.6; p=0.376] (control group mean: 84.9%) and 7.9 pp [95% CI: -10.4, 26.3; p=0.397 (reference group mean: 85.6%) respectively . When looking at condom use with all last three clients, these effects were 11.0 pp [95% CI: 0.8, 21.1; p=0.034] (control group mean: 67.5%) and 25.8 [95% CI: 5.2, 46.4] (reference group mean: 71.1%) respectively. "*

Main text

<u>Change 1: In Results, referred to SM Table with reference group means and removed PrEP referral control group means from main text regression results tables</u>

"Table 2 shows estimates of effects of PrEP referral, and the two measures of PrEP use on condom use and on perceptions of HIV and STI risks and reference group means are in ***SM Table S5****."*

Change 2: In Results, added reference group mean for PrEP use's estimand- Average treatment effect on the treated- and corrected interpretation

> *"The estimated effect of PrEP use on condom use with the last client is 7.9pp (95% CI: -10.4, 26.3; p=0.397). PrEP use was estimated to increase the probability of using a condom with all of the last three clients by 25.8 pp (95% CI: 5.2, 46.4; p=0.014) among PrEP users (reference group mean: 71.1%), which corresponds to the figure being 36.3% lower if PrEP users were not to have taken PrEP."*

Supplementary materials

Change 1: Add SM Table S5 with reference group means

Discussion

27. Can the author discuss potential contamination of the control group and its implications.

Response. The contamination of the control arm is a form of non-compliance. People in the treatment arm who do not take up PrEP is also a form of non-compliance. Very high non-compliance rates would mean that there is not a lot of difference between PrEP uptake rates between the treatment and the control arm. If PrEP uptake rates do not differ by much, then intuitively, we are unable to study our primary question of interest - how PrEP usage affects risky sexual behaviour- reliably by inducing a change in PrEP usage using PrEP referral (the experimental intervention). In our case, PrEP referral did increase PrEP uptake and exposure quite substantially (by 34.5pp and 39.1pp respectively), which enables us to study the behavioural response of PrEP usage. We added additional explanation of the latter in the Methods and Findings, Statistical analysis sub-section:

> *"We estimated effects of PrEP use and Ever used PrEP in past year on the outcomes. To estimate these effects allowing for non-random variation in use resulting from two-sided noncompliance, we used exogenous variation in each measure of PrEP use that was generated by random assignment to PrEP referral. That is, we used an encouragement design [26] in which assignment to the treatment or control group was effectively used as an instrument for PrEP use, ensuring identification of the effect from response of the respective outcome only to experiment-induced random variation in use."*

A second implication of non-compliance is its influence on statistical power as condom use in the overall sample is less likely to be observable. In the Limitations sub-section, we expand the discussion of contamination of the control group:

"… Furthermore, while study partners were asked to delay PrEP rollout in the control group until after the endline, this proved hard to implement. Nonetheless, randomised PrEP referral did substantially increase PrEP use, which made it possible to estimate the behavioural response, and we used statistical methods that ensured that this effect was identified only from the experiment-induced random variation in PrEP use."

Minor

28. Third paragraph in the section of study design and participants, methods, delete "a)"

Response. Thank you for catching the typo. We have edited this.

"We restricted the sample to survey participants who, based on information available in 2020, were potentially eligible for PrEP by excluding those who reported not doing sex work in 2020 or in any survey wave were recorded as having a public health system medical record showing they were HIV positive."

29. 2nd paragraph in the section of intervention, methods, it should be "experimental and control arms"

Response. Thank you for catching the typo. We have rephrased the sentence:

"At the end of that survey, treatment and control group participants who were still in sex work and reported being interested in receiving PrEP were referred for PrEP screening."

Reviewer #4

Important and interesting RCT looking at whether prep use causes risk compensation. This is important for understanding the full benefits of prep. The results are interesting although the high LTFU rate raised concerns, and it is a pity that the study relies on self-reported condom use which is a weak measure.

Response. Thank you for raising the concerns. We agree, and we have reordered the discussion of limitations to highlight these two important points first. We have also added emphasis of these limitations in the Abstract under Interpretation.

Comments

1. Great introduction to the paper laying out the existing knowledge.

Response. Thank you.

2. It was worrying to see the authors had limited data on aspects of the prep intervention, although it is unclear. Can you make it clearer what data was available for each arm and what was not.

Response. For both arms, we primarily relied on survey data. This includes PrEP data, data on condom use and other key behavioral outcomes, which were gathered for all participants. For PrEP uptake, we cross-checked with our partners wherever possible. For the treatment arm, we have incomplete information on the details of the PrEP initiation process (e.g. timings, why people fall out of the process, etc.) and no information on loss to follow-up from PrEP.

We have edited the main text in Methods, Procedures to make this clearer:

> *"As MoH and ANCS were responsible for PrEP implementation, for the treatment arm, we have limited and incomplete information on the losses at each stage of the recruitment, PrEP uptake and loss-to-follow up in the treatment arm, the reasons for these losses and the timings of each event, and no information on the control arm as we did not actively reach out to the control arm. Hence, the primary source of data for this analysis comes from the endline survey which was held in April-May 2022, where data from individuals in both arms was elicited."*

3. They say they checked self-reported prep use data - how well could that be done?

Response. In our study, we randomized FSWs into groups to receive pre-exposure prophylaxis (PrEP). At the time of the survey, participants were asked directly whether they were enrolled in PrEP as part of the trial. To ensure the accuracy of these self-reports, we

implemented a verification process. Midwives and FSWs' peer leaders who were involved in recruiting participants for the survey and had knowledge of their PrEP enrollment status were consulted. Since they knew participants, they confirmed whether each participant was effectively receiving PrEP at the health facility. This dual-check system ensured that our data on participants' PrEP status was both accurate and reliable, reducing the potential for misreporting or classification errors.

4. It is a pity the authors could not use a biological test or an anonymous method for condom use on the FSW because self-reported condom use is biased. This is something the study authors recognise and may have biased the prep users more if they were attending services more than the control arm. They note lower condom use in one of the anonymous methods - I assume they could not compare between prep and non prep users.

Response. Thank you for your thoughtful feedback. We acknowledge that the absence of biological outcome measures, such as STI diagnoses or the detection of prostate-specific antigen (PSA) or Y-chromosomal DNA, limits the robustness of our findings. However, we also note that while PSA and Y-chromosomal DNA have been used in prior studies (e.g., Giguère et al., 2019) to provide objective evidence of unprotected intercourse among FSWs, these methods also have some limitations. PSA detection provides only a short window of detection post-intercourse, and both markers may fail to account for differences in exposure based on frequency of sexual activity, condom slippage or breakage, and the impossibility to differentiate whether unprotected sex occurred with boyfriends or clients. Additionally, logistical and ethical challenges, as well as potential participant discomfort with biological sampling. Finally in the study of Giguère et al., 2019, note that there were discrepancies between PSA and Yc-DNA detection, where women tested positive for PSA but negative for Yc-DNA. We will expand our discussion to include these points and clarify the trade-offs associated with our reliance on self-reported data while acknowledging its inherent limitations. We have added this to the Limitations section on self-reported data:

> *"In this study, without biomarker outcomes, such as STI diagnoses or the detection of prostate-specific antigen (PSA) and Y-chromosomal DNA, it is not possible to test whether the finding that PrEP does not increase unprotected intercourse is robust to using a more objective measure of that outcome. While PSA and Y-chromosomal DNA have been utilized to measure unprotected sex, these methods also have limitations, including short detection windows, potential discrepancies between markers [21] and challenges arising from complications like condom slippage and distinguishing between sex with clients and boyfriends. Logistical, ethical and participant comfort considerations made it impractical to use biomarkers in this study setting."*

We did also attempt to collect condom use using two additional ‘anonymous’ methods. One of the methods (colorbox method) had the same overall level of condom use prevalence, hence we believe it would not have resolved any misreporting meaningfully. In this revision, we ran the results of this elicitation method for the RCT and will include it in the appendix. The other method used (list experiment) was elicited only for condom use with the last client. That particular outcome when directly elicited is already underpowered, and as the list experiment in a RCT context involves an experiment with noisy elicitation of condom use embedded within the broader PrEP experiment, it will be even more underpowered. Hence, we believe that the list experiment results are unreliable. In this revision, we generated the results using this method and will include it in the appendix, adding a note to mention our concerns about the reliability of this method.

> *“Second, the outcomes were primarily self-reported and may differ from actual behaviour due to intentional and unintentional misreporting that could result from social desirability bias and recall bias, respectively. If the degree of misreporting were to have differed between the treatment and control groups, it would bias estimated effects of PrEP. The bias would be upward if the treatment group were to overreport condom use to a greater extent. To assess this risk, in addition to directly asking participants about their use of condoms, we used two indirect elicitation methods. Although one of these methods gave a mean treatment effect estimate of condom use that was lower than the treatment effect obtained via direct elicitation, which is consistent with the latter being upwardly biased by a higher degree of overreporting in the treatment group, the main result of no negative effect of PrEP on condom use was found to be robust to measuring condom use with either method of indirect elicitation. There are two caveats on this robustness. First, the double list experiment may be unreliable because the method embedded an elicitation experiment within the PrEP experiment. This further reduced statistical power, which is the reason it was not used as the primary outcome measure. While the method reduced the risk of bias due to misreporting, it increased the risk that the null was not rejected because of low power and any statistically significant result has a higher likelihood of being a false positive. Second, the colorbox elicitation method gave estimates of condom use prevalence similar to those obtained with direct elicitation, which suggests that if the latter was prone to misreporting, the former method was not very effective in reducing the error. Future research should further investigate whether PrEP programmes influence incentives to misreport condom use.*

We agree that self report could potentially lead to an overestimation of condom use, especially among PrEP users who may be more engaged with healthcare services. We did not observe a reduction of other self-reported risky secondary outcomes that may have also been affected by increased engagement.

5. LTFU rates were high (only about 60% were surveyed at follow up_- what could the effect of that be? Did it occur more in the unregistered FSWs. Is it possible that those with higher LTFU had more indebtedness and so may have been more willing to use condom less while on PrEP.

Response. Regarding bias in terms of attrition, the main finding that we did not find any significant reduction in condom use remains robust to sample reweighting (we generate entropy balancing weights which tries to reweigh the final analysis sample's covariates to match the initial sample's moments). We include these results in SM Table S7.

6. I notice condom use decreased over the prep period by 10pp potentially due to decreases in condom use. Could there also be population optimism about HIV that results in decreases in prep users and non-users.

Response. Thank you for this insightful comment. We acknowledge the observed decrease in condom use over the PrEP period. We think the condom use decrease in our context due to population optimism is unlikely at the point of the evaluation survey as this may be an effect that takes time and PrEP has been just rolled out. However, we think that long-term equilibrium effects of PrEP usage among both PrEP users and non-PrEP users are definitely important to study as a broader shift in attitudes toward HIV risk, where participants feel more protected against HIV transmission due to PrEP could lead to less emphasis on condom use. Hence, your suggestion about potential population optimism regarding HIV risk- not just among PrEP users- is an important consideration. We originally mentioned this very briefly in the Limitations section regarding the point on long-term effects:

> *"There could also be community-level risk compensation of non-PrEP users when awareness of the prevalence of PrEP usage increases among FSWs [30]. Community-level risk compensation would invalidate RCT estimates unless properly accounted for."*

In this revision, to emphasise this point, we will include an additional paragraph on this in Limitations sub-section:

"In the longer term, broader population optimism about HIV risk due to the PrEP rollout may also be of concern and warrants further investigation. This would affect both PrEP users and

non-PrEP users as the population feel more protected and, as a result, place less emphasis on HIV prevention methods."

7. Prep use can be very transient in FSWs - what did the authors find in terms of retention on PrEP. Is it possible to get this data from their survey or the providers? I assume only long term users would think about reducing condom use, so maybe the follow up here is too short?

Response. Within the window of recruitment and the survey of a few months, we note that 20% of the people who started PrEP have already stopped using PrEP. Indeed this study can only study the short-term effects of PrEP usage. We also show that in the short-term, people's beliefs may not necessarily be correct. In reality, the marginal effect of using a condom over PrEP for HIV is in reality low if PrEP usage is consistent, while it does not seem to be what the respondents believe in as a whole. Hence, it is definitely possible that in the longer term, these beliefs would shift and users may trust PrEP more. Consequently, behaviour could change indeed. One important implication of this study from both the results and the experience in implementation is that risk compensation effects of PrEP may be substantial only in the long-term, but to study long-term effects, a RCT may be difficult. We emphasise this implication in the Limitations sub-section:

> *"Third, it was infeasible to estimate effects of PrEP on longer-term outcomes. Even though the overall HIV prevalence in Senegal is low (0.3%), among FSWs it is relatively high (6.6%) [17-18]. Hence, an extended delay in PrEP rollout in a control group would likely be considered unethical. Further, compliance in the treatment arm would likely decrease considerably over time, while contamination in the control arm would increase. Attrition from sex work and the survey would worsen with time. Short-term outcomes could differ from longer-term ones, especially since PrEP users among the study participants seemed to perceive that condom use provides substantial additional HIV protection on top of PrEP, even when PrEP should offer 99% protection against HIV when used consistently. Furthermore, the increasing awareness of clients about the introduction of PrEP in Senegal coupled with the low bargaining power of FSWs may reduce condom use in the longer term [11]. There could also be community-level risk compensation of non-PrEP users when awareness of the prevalence of PrEP usage increases among FSWs [30]. Community-level risk compensation would invalidate RCT estimates unless properly accounted for."*

8. Did an individuals perceived unlikelihood of getting HIV with Prep predict lower condom use in FSWs on PREP?
Response. We are unable to study this question using the randomized controlled trial's data per se because we had only elicited a perceived likelihood of getting HIV with PrEP among those who actually had used PrEP. While we could have posed it as a hypothetical question to non-PrEP users, subjective perceptions of PrEP and how these perceptions translate to a respondent's answer on the Likert scale could potentially be quite different among people using PrEP vs. people who have never used PrEP.

9. The authors state 'Anecdotally, a notable reduction in condom supplies of the MoH was observed after the introduction of PrEP, consistent with the increase in condom use found in the study.' Should it say decrease in condom use found in the study.
Response. To clarify, we meant an increase in condom use among PrEP users found in the study. MoH is one of the partners implementing PrEP provision. They provide free condom supplies at their facilities.
We edit the text in the Discussion sub-section:

> *"Anecdotally, a notable reduction in condom supplies of the MoH was observed after the introduction of PrEP, consistent with the increase in condom use among PrEP users found in the study."*

General editorial requests

(Note: not all will apply to your paper, but please check each item carefully)

1) We ask every co-author listed on the manuscript to fill in a contributing author statement, making sure to declare all competing interests. If any of the co-authors have not filled in the statement, we will remind them to do so when the paper is revised. If all statements are not completed in a timely fashion this could hold up the re-review process. If new competing interests are declared later in the revision process, this may also hold up the submission. Should there be a problem getting one of your co-authors to fill in a statement we will be in contact. Please do not add or remove authors without first discussing this with the handling editor. You can see our competing interests policy here: http://journals.plos.org/plosmedicine/s/competing-interests.
Response. We will comply with this.

2) Please upload any figures associated with your paper as individual TIF or EPS files with 300dpi resolution at resubmission; please read our figure guidelines for more information on our requirements: http://journals.plos.org/plosmedicine/s/figures. While revising your

submission, please upload your figure files to the PACE digital diagnostic tool, https://pacev2.apexcovantage.com/. PACE helps ensure that figures meet PLOS requirements. To use PACE, you must first register as a user. Then, login and navigate to the UPLOAD tab, where you will find detailed instructions on how to use the tool. If you encounter any issues or have any questions when using PACE, please email us at PLOSMedicine@plos.org.

Response. We will provide a new Fig1 image that has gone through this PACE diagnostic tool.

3) Please ensure that the paper adheres to the PLOS Data Availability Policy (see http://journals.plos.org/plosmedicine/s/data-availability), which requires that all data underlying the study's findings be provided in a repository or as Supporting Information. For data residing with a third party, authors are required to provide instructions with contact information (web or email address) for obtaining the data. Please note that a study author cannot be the contact person for the data. PLOS journals do not allow statements supported by "data not shown" or "unpublished results." For such statements, authors must provide supporting data or cite public sources that include it.

Response. The study data and code will be provided in a repository upon acceptance before publication.

4) We expect all researchers with submissions to PLOS in which author-generated code underpins the findings in the manuscript to make all author-generated code available without restrictions upon publication of the work. In cases where code is central to the manuscript, we may require the code to be made available as a condition of publication. Authors are responsible for ensuring that the code is reusable and well documented. Please make any custom code available, either as part of your data deposition or as a supplementary file. Please add a sentence to your data availability statement regarding any code used in the study, e.g. "The code used in the analysis is available from Github [URL] and archived in Zenodo [DOI link]" Please review our guidelines at https://journals.plos.org/plosmedicine/s/materials-software-and-code-sharing and ensure that your code is shared in a way that follows best practice and facilitates reproducibility and reuse. Because Github depositions can be readily changed or deleted, we encourage you to make a permanent DOI'd copy (e.g. in Zenodo) and provide the URL.

Response. We will make the code used in the analysis available at the point of publication together with the data in a public repository with a DOI link. We will edit the data availability statement and include the DOI link before publication after the paper is accepted.

5) FINANCIAL DISCLOSURES: The funding statement should include: specific grant numbers, initials of authors who received each award, URLs to sponsors' websites. Also, please state whether any sponsors or funders (other than the named authors) played any

role in study design, data collection and analysis, the decision to publish, or preparation of the manuscript. If they had no role in the research, include this sentence: “The funders had no role in study design, data collection and analysis, decision to publish, or preparation of the manuscript.”
Response. We have included this sentence in the text.

6) COMPETING INTEREST: All authors must declare their relevant competing interests per the PLOS policy, which can be seen here: https://journals.plos.org/plosmedicine/s/competing-interests
For authors with ties to industry, please indicate whether any of the interests has a financial stake in the results of the current study.
Response. We will comply to this.

7) DATA AVAILABILITY: The Data Availability Statement (DAS) requires revision. Since you have indicated that the data are accessible in the UCL repository database, please include the DOI or accession number.
Response. We will make data together with code used in the analysis available at the point of publication together with the data in a public repository with a DOI link. We will edit the data availability statement and include the DOI link before publication after the paper is accepted.

8) ETHICS: Please see the reviewer's comments regarding consent and clarify. Also, please indicate whether informed consent was written or oral.
Response. We have addressed the reviewer’s comments regarding consent and made proposed edits in the revision. We have indicated that survey consent is written.

FORMATTING - GENERAL
9) Abstract: Please structure your abstract using the PLOS Medicine headings (Background, Methods and Findings, Conclusions). Please combine the Methods and Findings sections into one section.
Response. We have relabelled the headings.

10) At this stage, we ask that you include a short, non-technical Author Summary of your research to make findings accessible to a wide audience that includes both scientists and non-scientists. The Author Summary should immediately follow the Abstract in your revised manuscript. This text is subject to editorial change and should be distinct from the scientific abstract. Ideally each sub-heading should contain 2-3 single sentence, concise bullet points containing the most salient points from your study. In the final bullet point of 'What Do These Findings Mean?', please include the main limitations of the study in non-technical language.

Please see our author guidelines for more information: https://journals.plos.org/plosmedicine/s/revising-your-manuscript#loc-author-summary.
Response. We have included this.

11) Please express the main results with 95% CIs as well as p values. When reporting p values please report as p<0.001 and where higher as the exact p value p=0.002, for example. Throughout, suggest reporting statistical information as follows to improve clarity for the reader "22% (95% CI [13%,28%]; p</=)". Please be sure to define all numerical values at first use.
Response. We have made these edits.

12) Please include page numbers and line numbers in the manuscript file. Use continuous line numbers (do not restart the numbering on each page).
Response. We have made these edits.

13) Please cite the reference numbers in square brackets. Citations should precede punctuation.
Response. We have done this.

FIGURES AND TABLES

14) Please provide titles and legends for all figures and tables (including those in Supporting Information files).
Response. We have done this.

15) Please define all abbreviations used in each figure/table (including those in Supporting Information files).
Response. We should have specified what abbreviations in each figure/table mean.

16) Please consider avoiding the use of red and green in order to make your figure more accessible to those with color blindness.
Response. We do not have coloured figures.

SUPPLEMENTARY MATERIAL

* Please note that supplementary material will be posted as supplied by the authors. Therefore, please amend it according to the relevant comments outlined here.

17) Please cite your Supporting Information as outlined here: https://journals.plos.org/plosmedicine/s/supporting-information
Response. We have complied with this

REFERENCES

18) PLOS uses the numbered citation (citation-sequence) method and first six authors, et al.
Response. We have complied with this.

19) Please ensure that journal name abbreviations match those found in the National Center for Biotechnology Information (NCBI) databases (http://www.ncbi.nlm.nih.gov/nlmcatalog/journals), and are appropriately formatted and capitalised.
Response. We have checked the journal names.

20) Where website addresses are cited, please include the complete URL and specify the date of access (e.g. [accessed: 12/06/2024]).
Response. We have complied with this.

21) Please also see https://journals.plos.org/plosmedicine/s/submission-guidelines#loc-references for further details on reference formatting.
Response. We have complied with this.

STUDY TYPE-SPECIFIC REQUESTS

22) PLOS Medicine requires that all trials be prospectively registered in one of registries recognized by WHO. Please ensure that study registration details are included in the Methods section.
Response. We have put the trial number in the text.

23) Please structure the Methods section using the following sub-headings: Study design and participants, Randomization and masking, Procedures, Outcomes, Statistical analysis.
Response. We have relabelled the headings.

24) The following outcomes measures appear to differ between the submitted manuscript and the protocol [and/or trial registry]. Please clarify and explain all discrepancies between the paper and protocol.

1) Primary outcome: Condom use with each of the last two clients and the last five clients measured via a combination of these methods: Via direct questioning, Via list experiment, Via a newly-designed instrument aimed at eliciting individual condom use without
2) Primary outcome: Subjective perceptions of the necessity of condom use measured using a Likert-like scale
3) Secondary outcome: Price of each of the last two clients
4) Secondary outcome: Earnings from sex work in a fixed time frame, e.g. 30 days

5) Secondary outcome: Household expenditure in a fixed time frame, e.g. 30 days
6) Secondary outcome: Food insecurity
7) Secondary outcome: Self-reported STI symptoms with last two clients
8) Secondary outcome: Mental health measured using the PHQ-9 questionnaire

Response.
1) Primary outcome: Condom use

Please see our response to the Reviewer 1 Comment 2 for the reason that we did not do the analyses on the list experiment and colourbox elicitation methods initially. We have performed these analyses and included it in SM Table S8 in this revision. We reference this table in the text.

2) Primary outcome: Subjective perceptions
This is a supporting outcome attempting to highlight a potential mechanism for the primary outcome on condom use. We originally included it as a secondary outcome in the initial manuscript due to its supporting role, but have put it back as a primary outcome following the reviewers' comments.
3) - 8) Secondary outcomes
We realized that the originally-specified secondary outcomes are unrelated to risky sexual behaviours, while the focus of our paper was risky sexual behaviours. Hence, we replaced these outcomes with survey questions that elicit aspects of risky sexual behaviours when writing the paper.

25) If the outcomes were not prespecified in the protocol, please define them in the Methods (Outcomes section) as post hoc and explain why they were added. Post-hoc comparisons should be presented as hypothesis generating rather than conclusive.
Response. We have complied with this.

> *"We included other aspects of risky sex behaviours other than condom use as post-hoc secondary outcomes."*

26) Please ensure that all prespecified outcomes (primary, secondary, and exploratory) are listed in the Methods/Outcomes section and indicate whether there are outcomes that are not presented in the current report.
Response. We have complied with this.

> **Abstract**
>
> *"Secondary outcomes defined post hoc were self-reported sexual risk taking, number of clients, proportion of regular clients, perceived HIV risk of clients, and type of sex act"*
>
> **Methods and Findings - Outcomes sub-section**

"Note that in the trial registration, we had originally specified secondary outcomes to be price of each of the last two clients, household expenditures and earnings from sex work in the last 30 days, food insecurity, self-reported STI symptoms with last two clients and mental health using the PHQ-9 questionnaire. However, we do not report these outcomes as the focus of the paper is on risky sexual behaviors. We replaced these outcomes with post-hoc secondary outcomes that better reflect risky sexual behaviors."

Conclusions - Results sub-section

"Table 3 shows estimates of effects of PrEP referral and PrEP use on post-hoc secondary outcomes on risky sexual behaviours."

27) Please specify the dates (Month Day, Year) during which study enrollment and follow up occurred.

Response. We do not know the exact day that the PrEP referral intervention started but it started in early September 2021. We indicate this in the text (*"From early September 2021 to January 2022"*). We indicate the dates of the follow up survey (*11 April to 13 May 2022*).

28) Please include absolute numbers wherever you report percentages; eg, n/N (%)

Response. We have complied with this.

29) Please present the safety data for the study including numbers of specific events and whether or not adverse events are thought to be related to treatment. AEs should be reported in the abstract, per CONSORT and CONSORT-Harms.

Response. There were no adverse events reported. We indicate this in the Procedures section.

"We received no reported adverse events from MoH and ANCS."

30) Please complete the CONSORT checklist (https://www.equator-network.org/reporting-guidelines/consort/) and ensure that all components of CONSORT are present in the manuscript, including how randomization was performed, allocation concealment, blinding of intervention, definition of lost to follow-up, power statement. When completing the checklist, please use section and paragraph numbers, rather than page numbers.

Response. We have completed the checklist.

31) Please report your abstract according to CONSORT for abstracts, following the PLOS Medicine abstract structure (Background, Methods and Findings, Conclusions) https://www.equator-network.org/reporting-guidelines/consort-abstracts/

Response. We have relabelled the abstract structure.

32) If your trial had to undergo important modifications in response to extenuating circumstances, please complete the CONSERVE-CONSORT checklist and provide in your Supporting Information; (https://www.equator-network.org/reporting-guidelines/guidelines-for-reporting-trial-protocols-and-completed-trials-modified-due-to-the-covid-19-pandemic-and-other-extenuating-circumstances-the-conserve-2021-statement/). When completing the checklist, please use section and paragraph numbers, rather than page numbers.
Response. Not applicable.

33) In keeping with our commitment to Open Science, please include the study protocol document and analysis plan (including any amendments) as Supporting Information to be published with the manuscript if accepted.
Response. The study protocol has been included as Supporting Information.

34) Please note that PLOS Medicine requires prospective, public registration of a data sharing plan (as part of mandatory clinical trials registration) for all clinical trials that began enrollment on or after January 1, 2019, in accordance with ICMJE requirements.
Response. Data and code will be shared via public repositories.
