# Supplementary material for: Effect of pre-exposure prophylaxis on risky sexual behaviour of female sex workers in Dakar, Senegal: A randomised controlled trial
Source: PLoS Med. 2025 Aug 18;22(8):e1004458. doi: 10.1371/journal.pmed.1004458 (PMC12407539; doi:10.1371/journal.pmed.1004458)
Supplement: Attachment — Submitted filename: PLOSMed Revision2 comments.docx [file pmed.1004458.s009.docx]

We thank the reviewers for giving further attention to our paper. We are extremely grateful to the editor for careful reading of the paper and detailed instructions on points to correct and clarify.

We have implemented all the requests for changes. Please find below a summary of the action taken in response to each comment.

Comments from Reviewers:

**Reviewer #4:** The author has thoughtfully responded to my comments. My only remaining comments are that it would be good to say how they added text in response to my comment 3 (about checking self reported data), and i still think the following sentence is not clear '"Anecdotally, a notable reduction in condom supplies of the MoH was observed after the introduction of PrEP, consistent with the increase in condom use among PrEP users found in the study'. Do they mean the MOH stocks of condoms reduced because of increased use of condom, or did they supply less each month but condom use still went up?

*Response*. We clarified the procedure used to check self-reported data on PrEP use as follows:

“These self-reports of PrEP utilisation were cross-checked, as far as possible, with MoH and ANCS records, and by asking midwives and FSW facilitators to confirm whether each participant was effectively receiving PrEP at a health facility.”

We have rewritten the quoted sentence as follows:

*“*Anecdotally, health ministry officials told the study team that more condoms were dispensed at public health centres after the introduction of PrEP, which is consistent with our finding that PrEP increased condom use.*”*

Requests from Editors:

GENERAL

*** In trials, there is usually a distinction in the language in terms of causal vs associational for primary and secondary trial outcomes. It would be beneficial to use associational language in the discussion and other sections for secondary outcomes.**

*Response.* We edited the text and tables to ensure consistency with this convention.

*** Please ensure that all abbreviations are defined at first use throughout the text (including statistical abbreviations). Please also check figures and tables.**

*Response*. Done.

*** Please review your text for claims of novelty or primacy (e.g. 'for the first time', ‘novel’) and remove this language.**

*Response*. Done.

*** Please check that any use of statistical terms (such as trend or significant) are supported by the data, and if not please remove them.**

*Response*. Done.

*** Please ensure that tables and figures, including those in supplementary files, are appropriately referenced in the main text.**

*Response.* Done.

*** Statistical reporting: Please revise throughout the manuscript, including tables and figures.**

**a) Please report statistical information as follows to improve clarity for the reader "22% (95% CI [13%,28%]; p</=)".**

*Response.* Done. We have not repeated the unit of measurement (% in the example above) in the CI since this seems unconventional and unnecessary.

**b) Please separate upper and lower bounds with commas instead of hyphens as the latter can be confused with reporting of negative values.**

*Response.* Done.

**c) Please define statistical definitions at first use and repeat the abbreviated definitions (HR, CI etc.) for each set of parentheses.**

*Response.* Done.

*** Please avoid referring to the study participants as sample. Please replace with cohort, participants or similar.**

*Response*. We have in the text replaced “sample” with either “cohort” or “participants.

*** We have noticed that in some tables the risk difference/effect estimate column is labeled as one or the other. Please review and use a consistent format.**

*Response.* We have now made clear in the text that we estimated (adjusted) risk differences (RDs) in primary outcomes and we have used this label in table titles and column headings. For non-binary secondary outcomes , we estimated mean differences (MDs). We use this label for these outcomes in table titles and column headings. At a few points in the text, we use “effect” as a generic label, but we do so only rarely.

*** When presenting results, we suggest not repeating numerical results with range and/or intervals in the main text if these results can also be found in the corresponding tables and figures. We think that this would streamline the text.**

*Response*. We have greatly reduced the number of numerical results from tables that we also present in the text. We have followed a rule of not repeating in the text numerical estimates that are not statistically significant. For main estimates that are significant, we do repeat some of them in the text in to guide the reader in interpreting the respectvie table and for emphasis. When we do this, we use the format proposed in the comment above: "X (95% CI [XX, XX]; p</=)".

*** In general, we feel that the main text, especially the results section, is not always easy to follow. Please revise carefully for clarity and conciseness, and be sure to guide the reader through your results.**

*Response.* We have edited the manuscript to improve clarity and make it more concise.

**DATA AVAILABILITY**

*** Please include the DOI or accession number.**

We have submitted the data set to UCL repository and are waiting for the DOI number. At the moment we can only share this link:

https://figshare.com/s/097817375bdeb43fe091

**ABSTRACT**

*** Please confirm that your abstract complies with our requirements, including providing all the information relevant to this study type** [**https://journals.plos.org/plosmedicine/s/submission-guidelines#loc-abstract**](https://journals.plos.org/plosmedicine/s/submission-guidelines#loc-abstract)

*Response*. We confirm that the abstract has been edited to comply with the requirements.

*** Per CONSORT, please note that only the primary outcome of the trial should be reported in your Abstract. Secondary outcomes should only be included in the Abstract if all secondary outcomes are fully reported. For trials that have many secondary outcomes, the Abstract should be limited to reporting the primary outcome.**

*Response.* We have removed all mentions of secondary outcomes.

*** In the last sentence of the Abstract Methods and Findings section, please describe the main limitation(s) of the study's methodology.**

*Response***.** We added**:**

“Main limitations were low power, high attrition, self-reported outcomes and a limited follow-up period.”

*** Please ensure that all numbers presented in the abstract are present and identical to numbers presented in the main manuscript text.**

*Response*: Done.

*** On line 44, we suggest writing ‘T’ and ‘C’ in full.**

*Response:* Done.

*** ll.46ff: Because you are describing percentage points, which describe a difference between two percentages, the estimated effect should be either increasing or decreasing. Please rephrase and be sure to indicate if the results are not significant.**

*Response.* We have revised the text as follows:

“Estimated effects of PrEP referral and PrEP use on condom use with the last client were positive but not statistically significantly different from zero. PrEP referral was estimated to increase the probability of condom use with all of the last three clients by 11.0 pp (95% CI [0.8, 21.2]; p=0.034). PrEP use was estimated to increase this probability by 25.8 pp (95% CI [5.2, 46.4]; p=0.014).”

With respect to the first sentence, please note that we prefer not to claim an increase and then say it is not significant, which would be inconsistent. We prefer the more neutral description of the direction of the estimated effects and their non-significance. Given the latter, we have dropped the numerical values of these estimates from the abstract.

*** Please note that you switch between "control group mean" and "reference mean". Do you always refer to the control group? If so, we recommend using the same description throughout.**

*Response.* We decided not to state relative estimates in tbe abstract because it appears not to be the norm in PLOS Medicine publications, and it avoids confusing the reader regarding the base used to calculate the relative change. In the main text and tables, when we do calculate relative effects, we no longer use the lavel “reference group mean” since it may confuse readers. In the statistical analysis section, we added an explanaton of the calculation of relative estimates:
*‘We also calculated this RD relative to the predicted outcome probability if there were no PrEP use, which was obtained by averaging predicted outcomes from the estimated bivariate probit under the counterfactual of no PrEP use.”*

In the results section, we now state clearly the base used for each relative estimate stated in the text. Table S6 presented the estimates of the counterfactual outcomes and provides further details on the calculation. When estimates are presented relative to the control group mean, we made this clear and show these measns in S5 Table.

*** We have noted that you discuss short-term effects, but have not provided details on time points or time lines between referral and self-reported outcomes. What was the average time between referral and endline survey?**

*Response.* We have clarified:

“We compared outcomes 3 to 8 months after referral of the treatment group and before referral of the control group.”

“This study, .. did not find evidence that PrEP referral or oral PrEP use increased self-reported risky sex behaviors of FSWs within 3 to 8 months.”

*** l.50: "There were no notable effects on other risky behaviours." - since this statement refers to the secondary outcomes, we suggest using associational language (if not removed, see second comment under Abstract).**

Response. *We have removed this statement.*

AUTHOR SUMMARY

*** We suggest changing the second bullet point to (split into two):**

**1) Risk compensation may be more pronounced among female sex workers (FSWs), as condomless sex is better remunerated.**

**2) According to a literature search in January 2024, there is a lack of randomised controlled trials testing whether PrEP uptake impacts the prevalence of unprotected sex among FSWs. For men who have sex with men, the evidence was mixed, with more recent studies showing risk compensation.**

*Response:* We have made the changes proposed.

*** We feel that the statement, “To our knowledge, this study provides the first evidence from a randomised experiment on whether PrEP causes compensatory risky sexual behaviour by FSWs.”, is somewhat repetitive of the statement on the literature search. We suggest removing it.**

*Response*. We have removed this sentence.

*** l.92: We suggest removing the word ‘strong’.**

*Response*. Done.

*** l.93: Again, we note that you discuss short-term effects, but have not provided details on time points or time lines between referral and self-reported outcomes.**

*Response*. We clarified.

*“We found no evidence that either PrEP referral or oral PrEP use reduces condom use or increases measures of sexual risk-taking within three to eight months.”*

*** ll.93-95: “The data are consistent with users of oral PrEP believing that combined protection with condoms is more effective than using PrEP alone.” – as written, it is not clear whether this statement reflects another finding of your study or whether it is based on recent literature.**

*Response.* We have rephrased to make clearer:

- *“* This may be because users of oral PrEP reported believing that PrEP is more effective when combined with condom use.”

*** l.98: “It is generally believed…” – is this based on scientific literature or scientific discourse among the research community?**

*Response.* We removed this sentence.

*** ll.100-103: We suggest creating a new bullet point for the statements starting “The evidence...” and ending “…in other setting”.**

*Response.* We have done this.

*** ll.104-107: Are there any indicators that the belief on combined PrEP and condom use is changing? We are not sure whether this statement is really needed here.**

*Response.* We have dropped this statement.

*** ll.107-109, we suggest changing to: Studies with longer follow-up are needed to evaluate behavioral responses. The study also demonstrates the difficulties of conducting a randomized controlled trial in this setting.**

*Response. We have dropped the claim about demonstrating the difficulties of conducting a RCT in the setting in order to make the message simpler.*

- *“The robustness of this result needs to be tested with larger samples and longer follow-ups in other settings.”*

*** Please note that the last bullet point should clearly state the main limitation(s).**

*Response***.** We added:

- “The study’s main limitations were low power that reduced the precision of estimates of effects on self-reported condom use, along with high attrition and a limited follow-up period.”

**INTRODUCTION**

*** Please change the heading to ‘Introduction’.**

*Response.* Done.

**METHODS AND RESULTS**

*** Please provide the approval numbers from both ethics committees.**

*Response:* We have added:

*“The study was approved by the National Ethics Committee for Research and Health Senegal (CNERS)* ***(No. 00000031)*** *and University College London Research Ethics Committee* ***(17341/001)****.”*

*** l.284: “sample of 500” – please change to: “We assumed a drop-out rate of 10% from the original study size of 500 individuals due to survey and sex work attrition,…”.**

*Response.* Done.

*** ll.351-353: Please remove the ‘Role of the funding source’ statement from the main text. These details should only be included in the metadata in the online submission form.**

*Response.* Done.

*** ll.336-337: “number of days in the seven preceding the endline interview that were within Ramadan” – please briefly explain the reason for adjusting for days within Ramadan.**

*Response.* We changed the text to:

*“number of days in the seven preceding the endline interview that were within Ramadan –* ***to account for the reduction in sex work during Ramadan – …****”*

*** Figure 1: Please define ‘PrEP’.**

*Response.* Done.

*** l.367: We suggest including the total n-number here for clarity (n=308).**

*Response.* Done.

*** l.382ff: Please clarify that these results refer to the total cohort.**

*Response.* Done.

*** ll.387-392: In what table/figure can readers find these values? Please provide a reference.**

**Throughout, we feel it needs to be more clear when results are not significant (cross the null).**

*Response.* We have added Table 2 that contains the statistics that were referred to here and revised the text accordingly.

We have edited the manuscript to make clear estimates that are not statistically significant at conventional levels.

*** l.394ff: We feel that it may be confusing to readers that you present risk differences (RD) in Table 2 while you continue to refer to "effect estimates" or "estimated effect" in the text. Also, in the table you present the risk difference as a decimal number, whereas in the text you present it as a percentage point. We feel that a consistent format (between tables/figures and text) would be preferable for the reader.**

***Response.***

We have now used risk difference (RD) consistently in the tables and text.

We have changed the RDs in tables to percentage points.

*** l.397ff: “The point estimate corresponds to a 3.8% increase over the reference (control) group mean (84.9%)” – We are not sure whether we missed it, but have you provided the outcome mean values for the intervention group anywhere (equal to S5 Table for the reference group)?**

*Response.* We have added S5 Table with mean values of the outcomes for the treatment and control groups.

*** l.424ff: “The signs of the point estimates are consistent with PrEP referral and use both strengthening beliefs that use of a condom is effective in preventing HIV, although the 95% confidence intervals all include zero.” – We think that this statement is not 100% clear and that the interpretation of the direction of risk differences should not be part of the results section.**

*Response***.** We dropped this sentence.

*** l.431: “using the full range of perceived HIV risks” – please re-phrase and be more specific.**

*Response.* We have rephrased this as:

*“*The estimates remain non-significant with a linear (1-5 scale) measure of HIV risk perceptions, *”*

In the Statistical analysis section, we refer to this alternative linear measure.

*** l.431: "The direction of these differences is robust..." - although the linear interpretation yielded similar directions, the results were not significant, i.e. we would suggest toning down these findings.**

*Response.* We change this to:

*“The estimates remain non-significant with a linear (1-5 scale) measure of HIV risk perceptions, although the p-values are smaller (S10 Table).”*

*** Tables: Please ensure to define all abbreviations used in tables and their descriptions, such as ‘PrEP’, ‘STI’, ‘CI’.**

*Response.* Done.

*** Table 1: Is there a reason why you decided to report Share of XYZ as decimal numbers instead of percentages?**

*Response.* We have changed it to percentages.

*** Table 2: In the figure description, you write “See S4 Table for estimates obtained using all values of these outcomes”. We are not 100% sure what the sentence means and also noted that S4 Table shows the characteristics of analysis cohort in 2020 and at endline (2022). Please clarify.**

*Response.*

We have rephrased as:

*“ See* ***S9 Table*** *for estimates obtained using alternative linear measures of these outcomes.”*

*** Table 3: “RDs were estimated as explained in notes to Table 2.” – please note that tables (and figures) should be self-explanatory on a standalone basis. Please repeat the explanation.**

*Response.* We have corrected this in the tables.

*** Table 3: “See S5 Table for estimates with alternative specifications and estimators” – please check whether this is a correct reference.**

*Response.* Thank you for catching the error. We have corrected it and checked the other references to SM material.

**DISCUSSION**

*** Please change the ‘Conclusions’ heading to ‘Discussion’.**

*Response.* Done.

*** Please remove any subheadings in the Discussion, including the Conclusion subheading.**

*Response.* Done.

*** l.491: Please note that in the discussion new results should not be presented. Please present the results on associations of risky sexual behaviours with PrEP use to the Results section (including the reference to S10 Table).**

*Response.* We have dropped this table and reference to the results in it.

------------------------------------------------------------

General Editorial Requests

**1) We ask every co-author listed on the manuscript to fill in a contributing author statement. If any of the co-authors have not filled in the statement, we will remind them to do so when the paper is revised. If all statements are not completed in a timely fashion this could hold up the re-review process. Should there be a problem getting one of your co-authors to fill in a statement we will be in contact. YOU MUST NOT ADD OR REMOVE AUTHORS UNLESS YOU HAVE ALERTED THE EDITOR HANDLING THE MANUSCRIPT TO THE CHANGE AND THEY SPECIFICALLY HAVE AGREED TO IT.**

We have not added or removed authors.

**2) Please ensure that the paper adheres to the PLOS Data Availability Policy (see** [**http://journals.plos.org/plosmedicine/s/data-availability**](http://journals.plos.org/plosmedicine/s/data-availability)**), which requires that all data underlying the study's findings be provided in a repository or as Supporting Information. For data residing with a third party, authors are required to provide instructions with contact information for obtaining the data. PLOS journals do not allow statements supported by "data not shown" or "unpublished results." For such statements, authors must provide supporting data or cite public sources that include it.**

All relevant data is available within the manuscript and Supporting Information files. Data have been also submitted to UCL repository.

**3) Please review your reference list to ensure that it is complete and correct. If you have cited papers that have been retracted, please include the rationale for doing so in the manuscript text, or remove these references and replace them with relevant current references. Any changes to the reference list should be mentioned in the rebuttal letter that accompanies your revised manuscript.**

*Response.* We have done this.

**Technical requests**

Journal Requirements:

**1) Please ensure that the funders and grant numbers match between the Financial Disclosure field and the Funding Information tab in your submission form. Note that the funders must be provided in the same order in**

**both places as well. Currently, your Funding Information is missing some of the grants and authors listed in your Financial Disclosure.**

*Response***.** We have amended this.

**2) Thank you for stating "No authors have competing interests"**

**If you have no competing interests to declare, please state "The authors have declared that no competing interests exist".**

*Response***.** We have changed this.

**3) Please ensure your short title is correct in the submission form, including symbols, italicisation etc. Your short title will be typeset from the submission form directly.**

*Response*. The short title is correct.

**4) We note that your MANUSCRIPT files are duplicated on your submission. Please remove any unnecessary or old files from your revision, and make sure that only those relevant to the current version of the manuscript are included.**

*Response***.** Duplicated files have been removed.

**5) We have noticed that you have uploaded " Trial protocole v2 (1).docx, CONSORT-2010-Checklist_july.docx, Supplementary Materials.docx, CONSORT-2010-Checklist_Revised.doc " files as supporting information but you have not included them in the list of legends. Please add a citation of this SI data files into the list of legends of your supporting information files.**

*Response.* We have included the Legend in the manuscript. Note that *CONSORT-2010-Checklist_july.docx* has been superseded by *CONSORT-2010-Checklist_Revised.doc*.

**6) Thank you for your statement " All files are available from the UCL repository database. "**

**In your Data Availability Statement, please ensure you have provided all necessary access information in complete sentences. Access information should include the name of any data repositories used as well as digital object identifiers (DOIs), accession numbers or codes, or other persistent identifiers for all relevant data. If all data has been provided within your submission files, please use the statement "All relevant data is available within the manuscript and Supporting Information files." The Data Availability Statement will appear on the published version of your manuscript.**

*Response***.** We have now submitted all those files within the submission.
