# Supplementary material for: Effect of pre-exposure prophylaxis on risky sexual behaviour of female sex workers in Dakar, Senegal: A randomised controlled trial
Source: PLoS Med. 2025 Aug 18;22(8):e1004458. doi: 10.1371/journal.pmed.1004458 (PMC12407539; doi:10.1371/journal.pmed.1004458)
Supplement: Attachment — Submitted filename: revisions made.docx [file pmed.1004458.s010.docx]

*Please upload the most recent version of the protocol as a Supporting Information file.

*We have done it.*

* Please note that you will need to provide a list of amendments as a Supporting Information file. Please confirm that these changes have been approved by an IRB and confirm that the approval was given prior to the data lock.

*We confirm that the changes to the prespecified outcomes were not submitted for additional IRB approval. However, the primary outcome changes—from "last five clients" to "last three clients"—were made prior to data lock and before any outcome data were accessed. These changes were made to improve the robustness and feasibility of measurement, and the rationale is detailed in the Supporting Information file.*

* We also need you to transparently explain in the Methods that the original protocol said X for primary outcomes, that the decision was made to change to Y, and that the changes were made prior to data lock. Please clarify if the changes were made before any survey questions were asked. As you have already done, please remember that it is important to direct readers to where you conducted additional analyses to show that the changes you made to your original plan did not substantially change the conclusions.

*We have now made the two following changes:*

*First:*

*“In supplementary analysis, we estimated the effect on condom use with the penultimate client, which was a prespecified outcome, and we used measures of condom use obtained from two indirect elicitation methods that aimed to reduce reporting errors.”*

*Change to:*

*“In supplementary analysis, we estimated effects on other prespecified outcomes: directly reported condom use with the penultimate client and measures of condom use elicited with two indirect methods that aimed to reduce reporting errors.”*

*Second:*

*“In the trial registration, we specified secondary outcomes to be price of each of the last two clients, household expenditures and earnings from sex work in the last 30 days, food insecurity, self-reported STI symptoms with last two clients and mental health using the Patient Health Questionnaire -9. We do not report these outcomes as our focus is on risky sexual behaviors. We replaced them with post-hoc secondary outcomes consistent with this focus.”*

*Change to:*

*“In the trial registration, the specified primary outcomes were directly reported condom use with the last client, the penultimate client and all of the last five clients, as well as condom use elicited with the two indirect methods and perceptions of risks of contracting HIV and STI. Prior to fielding the endline survey – and therefore before the data lock – we decided to reduce the survey burden by asking (directly) about condom use with the last three clients, not the last five. We did not seek ethical board approval for this change because it involved collecting less data. Pre-registered secondary outcomes included price charged to each of the last two clients, household expenditures and earnings from sex work in the last 30 days, food insecurity, self-reported STI symptoms with last two clients and mental health using the Patient Health Questionnaire -9. After data collection and unlocking of the data, we decided to replace these outcomes with the post-hoc secondary outcomes described above that better focus on risky sexual behaviors. We did not seek ethical board approval for this change because it had no consequence for the data collected.”*

* For reporting transparency, we encourage you to provide the 2025 SPIRIT checklist and ask you to update the CONSORT checklist to 2025 CONSORT. You can find the checklists here: <https://www.consort-spirit.org/>

*We have now updated the 2025 CONSORT and we have provided the 2025 SPIRIT checklist as well.*

Other editorial comments:

*Abstract, line 45, “308 (61.6%) were used for analysis”: We suggest changing to, “308 (61.6%) were included in the analysis”.

*We have changed this.*

*Abstract: Thank you for your clarification regarding the statement, " Estimated effects of PrEP referral and PrEP use on condom use with the last client were positive but not statistically significantly different from zero." We agree with the presentation of the results. If you wish, you may add the numerical results.

*We are not adding the numerical results on purpose since the results are not statistically significant.*

*Abstract: Please indicate that the analysis is a modified intention to treat (ITT) analysis.

*We have added this.*

*Please ensure to add a reference to the CONSORT checklist, the trial protocols and the list of amendments in the main text.

*We have added this.*

Additional requests:

1) Please ensure your short title is correct in the submission form, including symbols, italicisation etc. Your short title will be typeset from the submission form directly.

*The short title is correct.*

2) Thank you for your statement " All files are available from the UCL repository database." In your Data Availability Statement, please ensure you have provided all necessary access information in complete sentences. Access information should include the name of any data repositories used as well as digital object identifiers (DOIs), accession numbers or codes, or other persistent identifiers for all relevant data.

*The item is still under review and does not have a DOI yet for this reason. However, it can be cited as such and the DOI will be available on the following link:*

*Lepine, Aurelia (2025). PrEP randomised controlled trial among female sex workers, Senegal. University College London. Dataset.* [*https://rdr.ucl.ac.uk/articles/dataset/PrEP_randomised_controlled_trial_among_female_sex_workers_Senegal/28882685*](https://rdr.ucl.ac.uk/articles/dataset/PrEP_randomised_controlled_trial_among_female_sex_workers_Senegal/28882685)
